# Gene flow between island populations of the malaria mosquito, *Anopheles hinesorum*, may have contributed to the spread of divergent host preference phenotypes

**DOI:** 10.1111/eva.13288

**Published:** 2021-08-23

**Authors:** Luke Ambrose, Daniel Ortiz‐Barrientos, Robert D. Cooper, Neil F. Lobo, Thomas R. Burkot, Tanya L. Russell, Nigel W. Beebe

**Affiliations:** ^1^ School of Biological Sciences University of Queensland Brisbane Qld Australia; ^2^ Australian Defence Force, Malaria and Infectious Disease Institute Brisbane Qld Australia; ^3^ University of Notre Dame Notre Dame Indiana USA; ^4^ James Cook University Cairns Qld Australia; ^5^ CSIRO Brisbane Qld Australia

**Keywords:** gene flow, host preference evolution, island colonization, malaria, population genetics‐empirical

## Abstract

*Anopheles hinesorum* is a mosquito species with variable host preference. Throughout New Guinea and northern Australia, *An*. *hinesorum* feeds on humans (it is opportunistically anthropophagic) while in the south‐west Pacific's Solomon Archipelago, the species is abundant but has rarely been found biting humans (it is exclusively zoophagic in most populations). There are at least two divergent zoophagic (nonhuman biting) mitochondrial lineages of *An*. *hinesorum* in the Solomon Archipelago representing two independent dispersals. Since zoophagy is a derived (nonancestral) trait in this species, this leads to the question: has zoophagy evolved independently in these two populations? Or conversely: has nuclear gene flow or connectivity resulted in the transfer of zoophagy? Although we cannot conclusively answer this, we find close nuclear relationships between Solomon Archipelago populations indicating that recent nuclear gene flow has occurred between zoophagic populations from the divergent mitochondrial lineages. Recent work on isolated islands of the Western Province (Solomon Archipelago) has also revealed an anomalous, anthropophagic island population of *An*. *hinesorum*. We find a common shared mitochondrial haplotype between this Solomon Island population and another anthropophagic population from New Guinea. This finding suggests that there has been recent migration from New Guinea into the only known anthropophagic population from the Solomon Islands. Although currently localized to a few islands in the Western Province of the Solomon Archipelago, if anthropophagy presents a selective advantage, we may see *An*. *hinesorum* emerge as a new malaria vector in a region that is now working on malaria elimination.

## 1 INTRODUCTION

Host preference is a behaviour that varies between mosquito species and populations (Besansky et al., 2004; Takken, 1991; Takken & Verhulst, 2013) and is important in the epidemiology (Ritchie, 2014) of mosquito‐borne diseases (Clements, 2011; Hess et al., 1968; Lyimo & Ferguson, 2009; Zwiebel & Takken, 2004). Although many mosquito species are generalists (Takken & Verhulst, 2013; Tempelis, 1975) with a hierarchical preference for hosts (Hess et al., 1968), some mosquito species have a very strong preference for specific host types (Tempelis, 1975). Mosquito species with a strong preference for human hosts are the most efficient at spreading human disease. For example, *Anopheles gambiae* s.s. (the main vector of sub‐Saharan malaria in Africa (Sinka et al., 2010)) and *Aedes aegypti* (the main vector of dengue (Ritchie, 2014)) are preferentially anthropophagic (Costantini et al., 1999; McBride et al., 2014; Zwiebel & Takken, 2004). This means that they are more likely to take consecutive blood meals from humans than from different host species, increasing the survival and transmission of the pathogens that they transmit (Ritchie, 2014; White, 1974). Both *An*. *gambiae* s.s. and *Ae*. *aegypti* have evolved from generalists into human‐feeding specialists (Costantini et al., 1999; McBride, 2016), making them excellent models for studying the evolution of human host preference in mosquitoes (Brown et al., 2011; Carey & Carlson, 2011; Harrington et al., 2001; McBride et al., 2014).

In this study, we develop basic population genetic knowledge in a malaria‐transmitting species—*Anopheles hinesorum*—previously *An*. *farauti 2*, belonging to the *Anopheles farauti* complex. Although phylogeographic, behavioural and ecological studies have been performed on many members of this species complex (Ambrose et al., 2012; Beebe et al., 2000, 2015; Cooper et al., 2002; Van Den Hurk et al., 2000), it is as yet unstudied in terms of the basis of anthropophagy. The *An*. *farauti* complex is a particularly useful study system for elucidating the molecular basis of human host preference in mosquitoes due to differences in its host preference in geographically isolated populations and species (Beebe et al., 2015). *Anopheles hinesorum* is possibly the most useful species in the complex for studying the anthropophagy due to intraspecific differences in host preference.

*Anopheles hinesorum* has a wide distribution through much of the south‐west Pacific (Australia, New Guinea and the Solomon Archipelago) being found in coastal and inland habitats up to of over 1000 m above sea level (Beebe & Cooper, 2002; Beebe et al., 2015). Throughout most of its range, *An*. *hinesorum* is a host generalist, being opportunistically anthropophagic (Cooper et al., 2009; Keven et al., 2017; Laurent et al., 2017; Sweeney et al., 1990). However, most populations from the Solomon Archipelago do not bite humans (they are exclusively zoophagic; Beebe et al., 2000; Cooper & Frances, 2002; Foley et al., 1994). This is a well‐established phenotypic difference, and recent fieldwork (2015 and 2018) in Guadalcanal in Solomon Islands has further verified this finding where no *An*. *hinesorum* were collected in human landing catches (HLCs). These HLCs were performed near (within 50 m of) productive larval sites, no one has yet been able to collect blood fed adults from these populations, and their hosts remain unknown. A previous study also showed that exclusive zoophagy in *An*. *hinesorum* is a derived trait, finding two distinct zoophagic mitochondrial (mtDNA) lineages (Ambrose et al., 2012).

Ambrose et al. (2012) hypothesized that the evolution of exclusive zoophagy in these lineages may have occurred independently by convergent evolution. They found that the two lineages likely represent two separate dispersal events colonizing the Archipelago at different times in the past with the northern lineage representing an older dispersal event and the southern lineage representing a more recent dispersal event. In contrast to the hypothesis of convergent evolution of zoophagy, it is also possible that the initial (older) colonizing lineage had already adapted to feeding on local island hosts and that zoophagy was transferred from this preadapted population to the secondary (younger) colonizers via gene flow. Another possible scenario is that *An*. *hinesorum* colonized all islands in the Solomon Archipelago shortly after arriving there and that the secondary dispersal event to the southern islands resulted in the introduction and spread (via selective sweep) of a new mitochondrial lineage. Finally, it is possible that the initial population on the islands (presumably colonists from New Guinea or Australia) evolved or already exhibited zoophagy and contained multiple mitochondrial lineages which subsequently became dominant in the north and the south of the Archipelago.

As mentioned above, most populations of *An*. *hinesorum* in the Solomon Archipelago are exclusively zoophagic, including populations from Bougainville and Guadalcanal (Cooper & Frances, 2002; Foley et al., 1994). However, a recent study revealed anthropophagy in the Western Province Solomon Islands, where adult female *An*. *hinesorum* were collected landing (i.e. attempting to feed) on humans (Burkot et al., 2018). A few samples of the species have been collected on one other occasion in human landing catches on Santa Isabel, another island of the Solomon Archipelago, where it is very common in larval collections (Bugoro et al., 2011). Taken together, these studies show that there are behavioural differences in host preference between populations of this species within the Solomon Archipelago. The recently discovered anthropophagic population may have emerged as the result of the re‐evolution of anthropophagy from a zoophagic population. Alternatively, it may have been spread via gene flow from anthropophagic population(s) in Australia or New Guinea.

In this study, our first aim is to complement and build on previously published work with new nuclear microsatellite and mitochondrial data to better understand the population structure of *An*. *hinesorum*. This will lay the groundwork for its development as a novel model system for studying human host preference in mosquitoes. Our second aim is to use nuclear data to assess whether gene flow may have contributed to the spread of zoophagy between northern and southern island populations. Our third aim is to evaluate whether there is any evidence of gene flow from mainland Australia or New Guinea into the newly discovered anthropophagic *An*. *hinesorum* population in the Archipelago. To achieve these aims, we build on mitochondrial data (*n* = 233) published in Ambrose et al. (2012) to include additional Solomon Archipelago populations (*n* = 61). We develop 14 novel microsatellite primers for the species and generate microsatellite data from throughout the species range (*n* = 456). We include mitochondrial and nuclear microsatellite data from samples collected in human landing catches by Burkot et al. (2018), from the anthropophagic Western Province Solomon Islands population.

## 2 METHODS

### 2.1 Sampling and species identification

**FIGURE 1:**
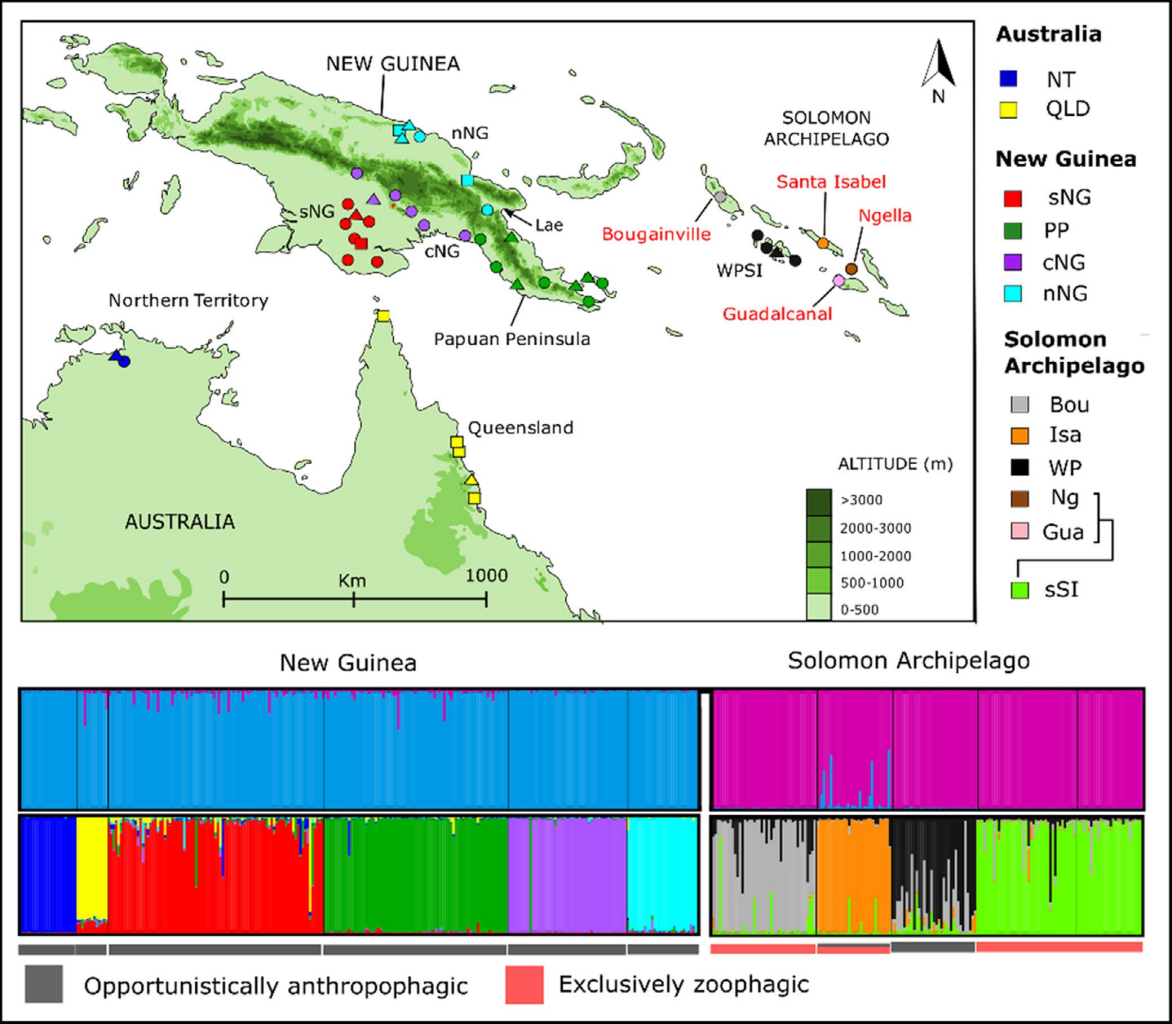
Map of sampling locations, host preference and Bayesian clustering (STRUCTURE) *Anopheles hinesorum* individuals based on 14 microsatellite loci. Host preference is shown by coloured bars beneath structure plots and by colour of text on the map. Opportunistic anthropophagic—feeds opportunistically on mammals including humans. Exclusively zoophagic—exclusively feeds on animals other than humans. Top Panel: Sampling locations of *An. hinesorum* specimens used in this study for microsatellite loci and mitochondrial *COI*. For some sites, only *COI* was generated/available (represented by squares); for others, only microsatellites were generated (represented by triangles), circles show sites for which both *COI* and microsatellite data are available. Locations labelled in red represent populations that do not bite humans, while populations labelled in black represent populations that are anthropophagic opportunists. The location of the city of Lae referred to in the manuscript is shown by the black arrow. Sites are coloured by the genetic population/cluster as follows: NT—Northern Territory; QLD—Queensland; sNG—southern New Guinea; PP—Papuan Peninsula; cNG—central New Guinea; nNG—northern New Guinea; Bou—Bougainville; Isa—Santa Isabel; WP—Western Province Solomon Islands; (Gua—Guadalcanal plus Ng—Nggela Islands) = sSI (southern Solomon Islands). Bottom Panel: The plots above were generated using the program STRUCTURE. They show individual membership probabilities to each population. The STRUCTURE plots represent the major clusters found (*K *= 2 and *K *= 10) in STRUCTURE analyses. For further details on these analyses, see the Section 2

Specimens for this study were collected as both larvae and adults, with some samples collected in human landing catches (Table 1 and Figure 1). Genomic DNA was isolated, and samples were verified as being *An*. *hinesorum* using a well‐established PCR diagnostic method (Beebe & Saul, 1995).

**TABLE 1:** Summary of *Anopheles hinesorum* collections and of the number of individuals genotyped for nuclear microsatellites and mtDNA COI

Region	Population	Site code	Collection	*n* (msats)	*n* (COI)	Coordinates
Aus	Qld	1	T	0	8	−18.216, 145.854
		2	T	13	0	−17.457, 145.859
		3	T	0	0	−17.409, 145.947
		4	T	0	6	−16.674, 145.328
		5	T	0	7	−16.252, 145.302
		6	T	0	19	−10.954, 142.462
	NT	7	T	15	7	−12.949, 132.549
		8	T	8	4	−12.76, 132.66
PNG	sNG	9	T	15	9	−7.729, 141.490
		10	T	11	6	−7.079, 141.989
		11	T	20	0	−7.011, 141.490
		12	T	15	5	−7.182, 141.248
		13	T	16	10	−6.824, 141.372
		14	T	4	0	−7.319, 144.179
		15	T	4	3	−8.622, 141.137
		16	T	4	1	−8.635, 142.219
		17	T	0	5	−8.074, 141.750
	cNG	18	T	19	11	−7.865, 145.669
		19	T	16	11	−6.781, 143.469
		20	T	8	0	−7.799, 146.443
		21	T	2	0	−6.295, 142.234
		22	T	4	1	−6.205, 143.011
	PP	23	T	12	9	−9.3485, 147.1894
		24	T	19	8	−7.966, 146.214
		25	T	9	12	−9.668, 150.787
		26	T	8	0	−9.629, 147.551
		27	L	4	4	−10.329, 150.272
		28	L	4	0	−9.629, 147.551
		29	L	4	0	−7.879, 147.141
		30	L	5	4	−9.506, 148.462
		31	T	5	0	−9.704, 149.652
		32	T	6	0	−9.312, 150.322
	nNG	33	T	4	4	−6.861, 146.351
		34	T	0	1	−5.769, 145.592
		35	T	15	0	−4.069, 143.256
		36	T	6	3	−3.782, 143.366
		37	T	4	1	−3.956, 143.927
		38	HLC	0	5	−3.80, 143.066
SI	Bou	39	L	43	23	Bougainville
	Isa	40	L, HLC	31	27	Santa Isabel
	WP	41	HLC	8	0	Nazareth
		42	L	22	20	Ghizo
		43	HLC	0	4	Tuguivili
		44	HLC	0	2	New Mala
		45	HLC	5	0	Kinamara
	Gua	46	L	41	21	Guadalcanal
	Ng	47	L	27	5	Nggela Islands
Total				456	266	

Note

Coordinates are provided in latitude and longitude in decimal degrees (WGS84 geodetic datum).

Abbreviations: cNG, central Papua New Guinea; HLC, human landing collection; L, larval collections; nNG, northern New Guinea; NT, Northern Territory Australia; PP, Papuan Peninsula; Qld, Queensland Australia; sNG, southern Papua New Guinea; T, CO2 CDC trap collection.

### 2.2 Mitochondrial sequencing and analysis

We sequenced, edited and aligned a 527 base‐pair sequence of the mitochondrial cytochrome oxidase 1 gene (mtDNA *COI*) for 60 individuals in this study. We aligned this with previously published homologous sequence data (*n* = 206; Ambrose et al., 2012) for further analysis. The new data include 27 individuals from Santa Isabel Island (including eight adult females caught in human landing catches in a previous study; Bugoro et al., 2011), 26 samples from the Western Province of the Solomon Islands (see Table 1), five individuals (larvae) from the Nggela Islands and two additional individuals (larvae) from Bougainville Island. Of the 26 individuals collected from Solomon Islands Western Province, 16 were adult females (collected biting humans) and 10 were collected as larvae. We generated data for this study using the same primers and methods outlined in Ambrose et al. (2012) and then edited and realigned them to the pre‐existing *COI* alignment in the program Geneious v.8 (Kearse et al., 2012). To assess relationships between populations, we generated a median joining mitochondrial haplotype network using the program PopART (Bandelt et al., 1999).

### 2.3 Microsatellite development and scoring

We developed 14 novel microsatellite markers using the same methods described in Ambrose et al. (2014). We called fragment sizes manually using the program, GeneMarker v.2.2 (Hulce et al., 2011) and removed individuals missing data from six or more loci from the data set prior to analysis, leaving 456 individuals in the final data set. We initially defined populations based on genetically distinct groups identified by Ambrose et al. (2012), and we treated separate islands in the Solomon Archipelago as populations. We then checked for the presence of null alleles using MicroChecker v2.2.3 (Van Oosterhout et al., 2004) and for Hardy–Weinberg equilibrium (HWE) in the R package *PopGenReport* (Adamack & Gruber, 2014). For primer‐ and locus‐specific information, including information on null alleles and HWE, see Table S1.

### 2.4 Microsatellite population structure

We performed a variety of analyses to assess population structure of *An*. *hinesorum* throughout its range based on nuclear microsatellite data. These include Bayesian analyses (STRUCTURE), multivariate analyses, estimation of fixation indices, a neighbour‐joining tree based on pairwise *G*′^ST^ and AMOVA. Initially, we assessed population structure using the Bayesian clustering program STRUCTURE v. 2.3.4 (Pritchard et al., 2000). We ran STRUCTURE through the program STRUCTURE_threader (Pina‐Martins et al., 2017) for 20 iterations of *K *= 2 to *K *= 15, using the admixture model and location priors (100,000 generation burn‐in, 500,000 generation sampling). Sites where mosquitoes were sampled were used to define location priors for populations from Australia and New Guinea. In the Solomon Archipelago, we used the islands that individuals were sampled from as location priors. We ran STRUCTURE output through the CLUMPAK server (Kopelman et al., 2015), with default CLUMPP (Jakobsson & Rosenberg, 2007) and DISTRUCT (Rosenberg, 2004) settings, including the LargeKGreedy algorithm (in CLUMPP), with a random order of input and 2000 repeats. We determined the most strongly defined population structure in the data using CLUMPAK which implements the Evanno delta *K* method (Evanno et al., 2005) as well the most probable *K* based on the ‘Estimated Ln Prob of Data’ (Kopelman et al., 2015). As has been found previously, the Evanno method underestimated the optimal value of *K* (Janes et al., 2017). We therefore present the major mode for STRUCTURE plots for both *K *= 2 (predicted by the Evanno method) and *K *= 10 (predicted by the ‘Estimated Ln Prob of Data’). Additional STRUCTURE plots for all *K* values run can be found in Data S1.

We also used three multivariate clustering methods—principal components analysis (PCA), discriminant analysis of principal components (DAPC; Jombart et al., 2010) and t‐distributed stochastic neighbour embedding (t‐SNE; Van Der Maaten & Hinton, 2008)—to assess population structure. T‐distributed Stochastic Neighbour Embedding is a multivariate method based on machine learning that is used to visualise multidimensional data in two or three dimensions. It is similar in concept to principal component analysis in that it arranges points (representing individuals) in space such that highly similar points are located close together (clustered) while dissimilar points are dispersed (Van Der Maaten & Hinton, 2008). An advantage of these multivariate approaches is that they are free of population genetic assumptions; for example, there is no assumption that populations are in HWE. We performed both PCA and DAPC analyses in the *adegenet* package (Jombart, 2008) and the t‐SNE analysis in the *Rtsne* package (Krijthe, 2015) in R version 3.3.0 (R Core Team, 2013), run through RStudio version 1.0.136 (Rstudio Team, 2020). For these analyses, we replaced missing data with mean values for the overall data.

We initially defined populations (as requested by DAPC) based on results by Ambrose et al. (2012) as well as results from initial STRUCTURE analyses. For DAPC analyses, we used the cross‐validation *xvalDAPC* command with 1000 replicates to determine the optimal number of principle components (PCs) to retain for each analysis. For the full data set, we retained 80 PCs and ten discriminant axes (DAs), and for the Solomon Archipelago data alone (*n* = 177), we retained 30 PCs and 4 DAs. We present two types of plots that were generated from the DAPC: a composition plot (a bar plot—similar to a STRUCTURE plot) and pairwise plots of the first two discriminant axes.

We used the program GenAlEx v.6.5 (Peakall & Smouse, 2006) to estimate pairwise fixation indices, *G* ^ST^, *G*′^ST^ and Jost's D, between the populations identified by STRUCTURE and multivariate methods. We plotted results for one of these indices (*G*′^ST^) in tabular form, as well as building a neighbour‐joining tree based on pairwise *G*′^ST^ using the R package *ape* (Paradis & Schliep, 2019). Finally, we performed an AMOVA (Excoffier et al., 1992) to partition variance explained by different hierarchical strata in the data. To achieve this, we used the *poppr*.*amova* function implemented in the R package *poppr* (Kamvar et al., 2014). Prior to running the AMOVA, we defined strata within our data by region (Australia, New Guinea, Solomon Archipelago) as well as by populations identified by STRUCTURE and multivariate analyses. We then performed a randomized test using the *randtest* function to assess whether there is significantly more or less variance explained by different partitions (strata) in the data compared with the null (random) expectation.

## 3 RESULTS

### 3.1 Mitochondrial DNA genetic structure

**FIGURE 2:**
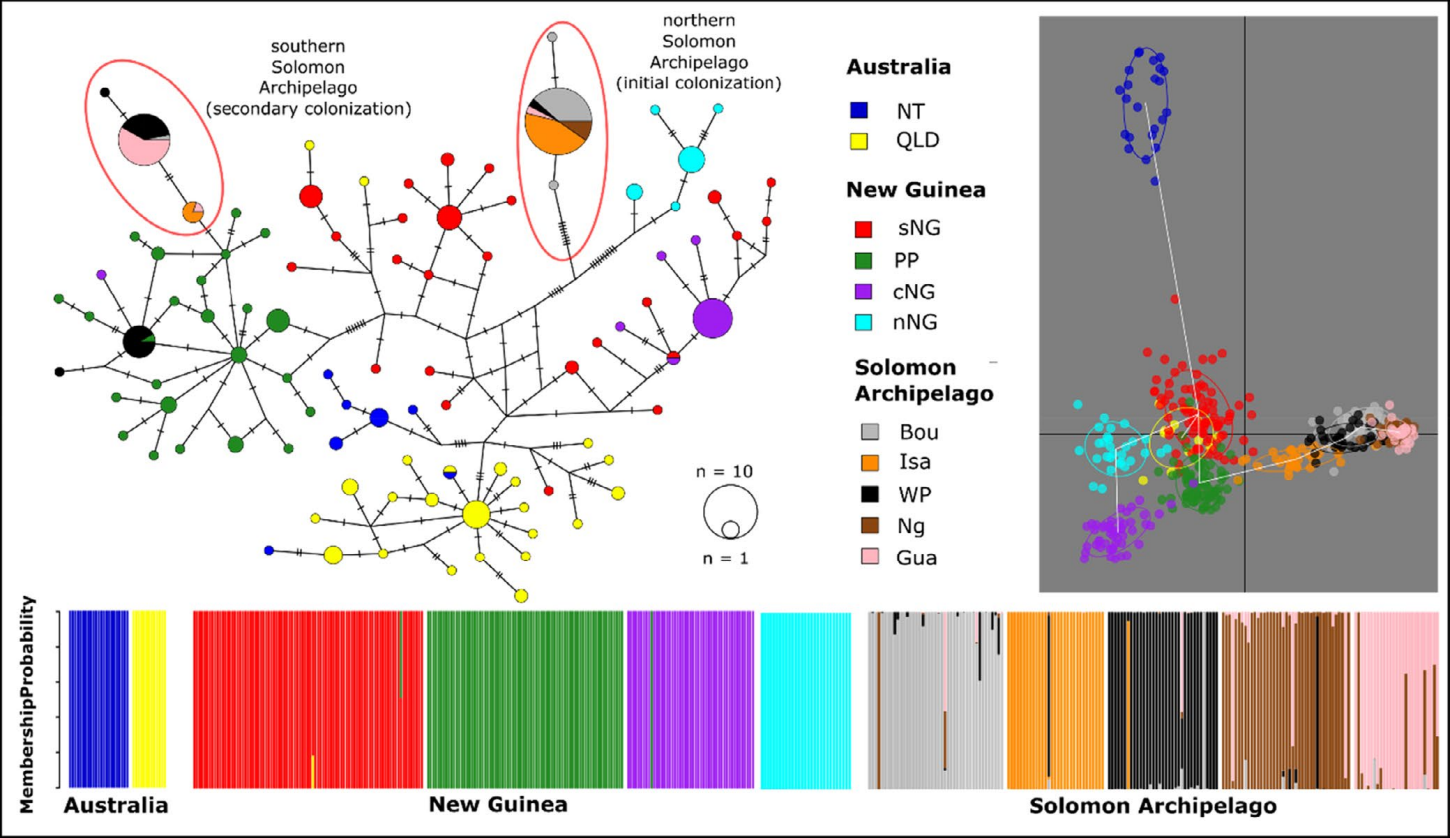
Median joining haplotype network of *Anopheles hinesorum* mitochondrial *COI* sequences and DAPC analysis. Top Left: Relationships between mitochondrial haplotypes from 527 bp of the cytochrome oxidase 1 gene. Each circle represents a unique sequence with lines between sequences and hatches crossing lines showing mutations. Circles representing haplotypes are coloured by geographical region as shown in the key at bottom right and the size of each circle and proportion coloured shows the number of individuals that were sampled with that haplotype. Red circles show the two distinct lineages that are found on the Solomon Islands. Top Right: DAPC—scatter plot of all *An. hinesorum* microsatellite data generated. Each point represents a single individual and distance between points is negatively correlated with how closely related they are. Individuals are coloured by population as shown in the key above. The white line connecting groups is an estimated minimum spanning tree. Bottom: DAPC—Individual assignment plot generated using all *An. hinesorum* microsatellite. It shows the probability of assignment of each individual to a given population. Abbreviations are as follows: cNG, central New Guinea; Gua, Guadalcanal; Isa, Santa Isabel; Ng, Ngella Islands; nNG, northern New Guinea; nSI, northern Solomon Archipelago; NT, Northern Territory; PP, Papuan Peninsula; QLD, Queensland; sNG, southern New Guinea; WP, Western Province Solomon Islands

The mtDNA haplotype network (Figure 2) expands on previously published work (Ambrose et al., 2012), with the addition of samples from the Solomon Islands Western Province, Santa Isabel and the Nggela Islands and new samples from Bougainville. We found three major patterns: firstly, we observed higher haplotype diversity in Solomon Islands Western Province than on the other islands in the Archipelago—six unique haplotypes were sampled from the 26 individuals from the Solomon Islands Western Province. Three of the haplotypes sampled from this population are identical to haplotypes sampled elsewhere in the Solomon Archipelago (two from the more recent southern lineage and one from the older northern lineage). One haplotype belongs to the recent southern lineage but has not been sampled elsewhere. Secondly, for the first time, we found haplotype sharing between the Solomon archipelago and a New Guinean population, with two haplotypes sampled from Western Province falling within a group from the Papuan Peninsula of eastern Papua New Guinea. One of these haplotypes was commonly sampled from Western Province (*n* = 11, six adult females and five larvae from Ghizo Island) and is shared with individuals from the Papuan peninsula. The other haplotype falling in this group was only sampled in one larva collected from Ghizo. Thirdly, apart from Western Province, all newly sampled Solomon Archipelago *COI* haplotypes belong to the northern island lineage.

### 3.2 Microsatellite analyses

**FIGURE 3:**
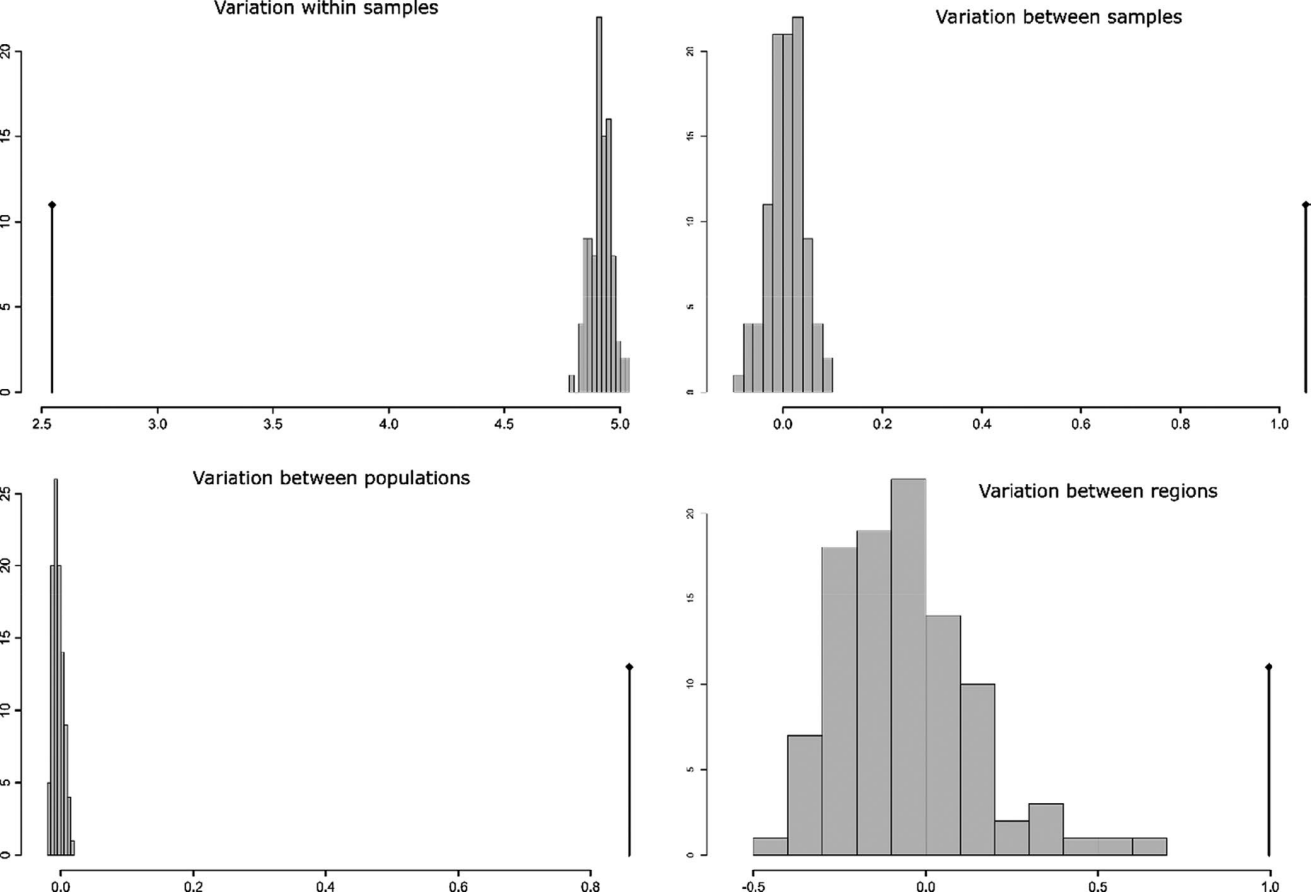
Null (randomised) distribution versus observed variance explained by different strata in AMOVA analyses. The figure above shows observed (black line) variance (Sigma) versus the null expected distribution, which was generated by randomisation of data in R, as outlined in Excoffier et al. (1992). The top left panel shows that the observed Sigma within samples is lower than expected under the null distribution (*p* < 0.01). The top right panel shows that Sigma observed between samples within populations is higher than expected (*p* < 0.01). The bottom left panel shows that Sigma observed between populations within regions is higher than expected (*p* < 0.01). The bottom right panel shows that Sigma observed between regions higher than expected (*p* < 0.01)

All microsatellite analyses identified strong genetic structure between populations defined a priori. We find support for all previous genetic groups found by Ambrose et al. (2012) with a high probability of assignment to a single cluster for most individuals in both DAPC and STRUCTURE analyses (Figures 1 and 2). At *K *= 2, STRUCTURE separated individuals from the Solomon Islands from individuals sampled from Australia and New Guinea, which form a single group. At *K *= 10, STRUCTURE identified two groups in Australia (Northern Territory and Queensland), four in New Guinea (southern New Guinea, Papuan Peninsula, central New Guinea and northern New Guinea) and four in the Solomon Archipelago (northern Solomon Islands, Santa Isabel, Solomon Islands Western Province and southern Solomon Islands). DAPC analysis revealed evidence of eleven distinct clusters, in agreement with those found by STRUCTURE but with additional genetic differentiation between Guadalcanal and Nggela (which form the southern Solomon Islands indicated above; Figure 2). For results from STRUCTURE analyses from *K *= 2 to *K *= 15, see Data S1. AMOVA showed significantly higher than expected levels of variance between samples within populations (phi = 0.29, *p* = 0.01), between populations within regions (phi = 0.19, *p *= 0.01) and between regions (phi = 0.18, *p *= 0.01) and significantly lower than expected variance within samples (phi = 0.53, *p* = 0.01) compared with the null model (Figure 3 and Table 2).

**TABLE 2:** Results from AMOVA analyses

	*df*	Sums of squares	Mean square	Sigma	%	Phi	*p*
Between regions	2	641.79	320.89	1.0	18.26	0.18	0.01
Between populations within region	8	604.61	75.58	0.86	15.75	0.19	0.01
Between samples within population	445	2069.34	4.65	1.05	19.31	0.29	0.01
Within samples	456	1160.5	2.54	2.54	46.68	0.53	0.01

Note

This table shows results of AMOVA analyses. *df* = degrees of freedom, Sigma = variation explained by each level, % = the percentage of total variation explained by each level, Phi = the Phi statistic for each level. *p*‐values were obtained by permutation tests as described in Excoffier et al. (1992).

**FIGURE 4:**
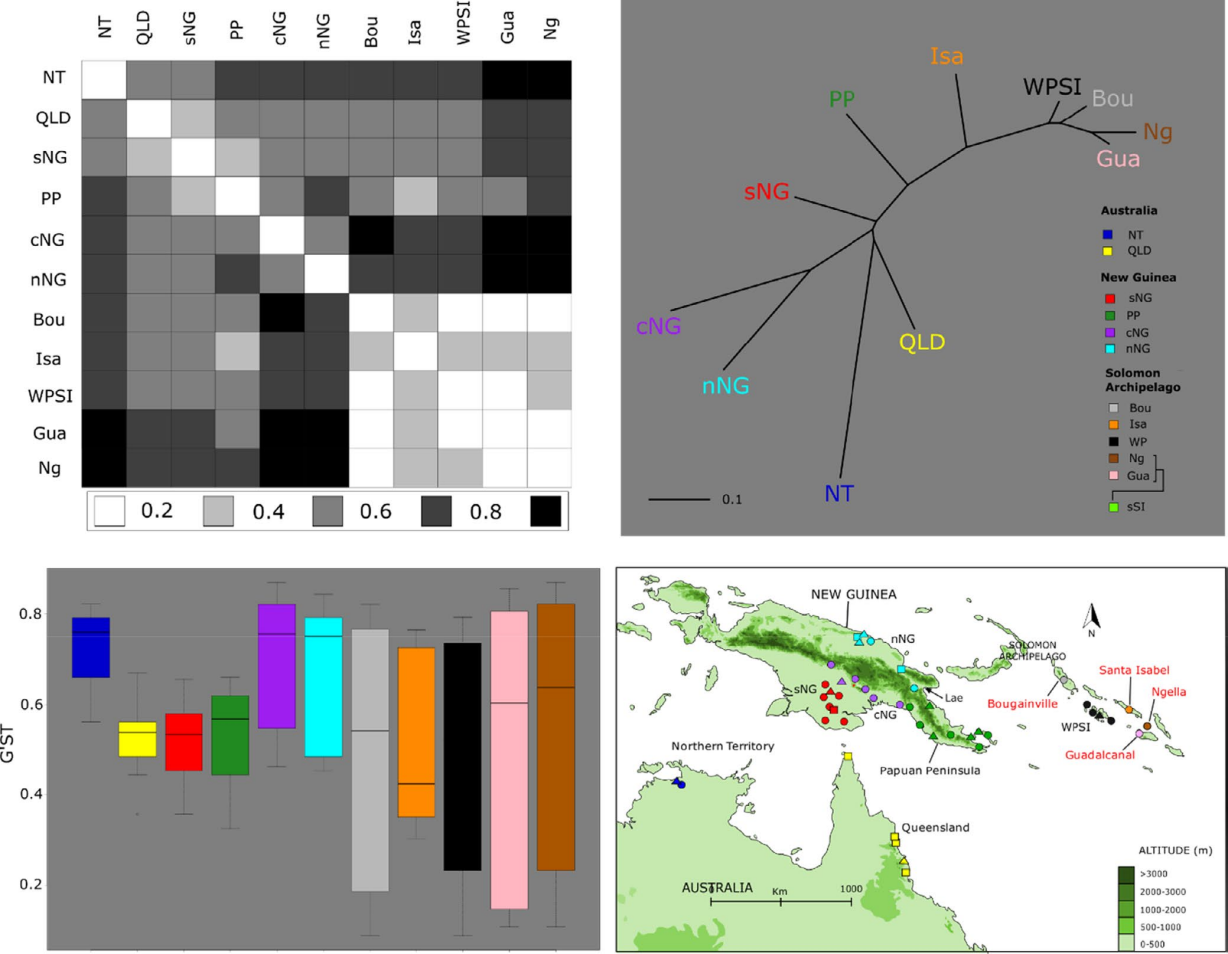
Summary of *Anopheles hinesorum* population structure based on pairwise *G*′^ST^ (14 nuclear microsatellite loci). Top Left: Graphical representation of pairwise *G*′^ST^ values between *An. hinesorum* populations based on allele frequencies of 14 microsatellite loci. Values shown in key are pairwise *G*′^ST^ values calculated in GenAlex. Top Right: Neighbour joining tree showing relationships between populations based on pairwise *G*′^ST^. Bottom Left: Box plots showing ranges and interquartile ranges of pairwise *G*′^ST^ values for each population. Bottom Right: Map of sampling locations with sites coloured by population. Abbreviations are as follows: cNG, central New Guinea; Gua, Guadalcanal; Isa, Santa Isabel; Ng, Ngella Islands; nNG, northern New Guinea; nSI, northern Solomon Archipelago; NT, Northern Territory; PP, Papuan Peninsula; QLD, Queensland; sNG, southern New Guinea; WP, Western Province Solomon Islands

The Northern Territory (Australia) population is the most distantly related at the nuclear level based on consistently high pairwise fixation indices relative to the other populations (Figure 4). Within New Guinea, the northern and central populations are the most distinct for microsatellite loci, also based on pairwise fixation indices (Figure 4 and Tables S2–S4). Despite strong population structure within New Guinea, one individual from the central New Guinean population falls within the Papuan peninsula group with mtDNA and the microsatellite analyses (Figures 1 and 2). Microsatellite markers also show a close relationship between southern New Guinean and Queensland populations (Tables S2–S4, Figure 4), which form the centre of the multivariate plots (Figure 2 and Figure S1). Despite this close relationship, STRUCTURE, DAPC and t‐SNE show that the Queensland population constitutes a separate group (Figures 1 and 2 and Figure S1).

The Solomon Archipelago populations cluster together in the multivariate analyses to form a closely related group that is distinct from populations from Australia and New Guinea. The STRUCTURE analysis run at *K*2 also clearly separates the Solomon Archipelago from populations from Australia and New Guinea (Figure 1). This taken together with low fixation indices between island populations relative to the overall average *G*′^ST^ suggests that there has been recent nuclear gene flow through the Archipelago. In assessing the relationship between mainland and island populations, the population from the Papuan Peninsula (New Guinea) and Santa Isabel (Solomon Islands) appear as closest relatives in the DAPC (Figure 2) as well as in pairwise *G*′^ST^ estimates (Figure 4).

Within the Solomon Archipelago, nuclear divergence based on pairwise measures of differentiation is consistently lowest between the southern, western and northern Solomon Archipelago populations, with Santa Isabel being the most distinct population in this region based on pairwise fixation indices (Figure 4 and Tables S2–S4). Populations from Nggela and Guadalcanal are closely related, with evidence of weak genetic structure between these islands in multivariate analyses (Figure 2 and Figure S2). The close relationship between these populations is further verified by relatively low fixation indices between these populations (Figure 4 and Tables S2–S4). All analyses also suggest a close nuclear genetic relationship between the Bougainville and Solomon Islands Western Province populations.

## 4 DISCUSSION

### 4.1 Population structure between mainland *An. hinesorum* populations

By expanding field collections and using nuclear microsatellites, we build on previous knowledge of the population structure of *An*. *hinesorum* in the south‐west Pacific. Within New Guinea, we find six genetic clusters corresponding to geographically restricted populations identified in a previous study (Ambrose et al., 2012). Pairwise fixation indices based on microsatellite data corroborate previous findings, showing that the northern New Guinean population is the most genetically distinct population in New Guinea. DNA sequences of ribosomal protein S9 DNA from individuals sampled from the northern New Guinean population were previously found to be completely sorted from other populations of the species, forming a well‐supported monophyletic clade (Ambrose et al., 2012). The northern New Guinean population was also the only *An*. *hinesorum* population that could not be detected by species‐specific genomic DNA probes (Beebe et al., 1996). Altogether, this evidence suggests putative cryptic species status. However, further evidence of reproductive isolation when in sympatry with other *An*. *hinesorum* populations, or other genomic evidence of reproductive isolation would be necessary to make this assessment. There may be areas of parapatry where the ranges of the northern New Guinean and the Papua Peninsula populations meet—inland from the city of Lae—where reproductive isolation could be assessed.

Other populations in New Guinea that are found in proximity to each other are the central New Guinean, southern New Guinean and Papuan peninsula populations. The southern and central New Guinean populations as well as the central New Guinean and Papuan peninsula populations may have areas of parapatry on the southern New Guinean coast (see Figure 1). Despite this, the central New Guinea population is distantly related in microsatellite analyses and is completely sorted for rpS9 (Ambrose et al., 2012), but shares mtDNA haplotypes with the highly diverse southern New Guinean population. One individual sampled from a site in the Papuan Peninsula, bordering the central New Guinean population range, was assigned to the central New Guinean population in microsatellite analyses with strong support and no evidence of admixture. These results suggest that populations identified within New Guinea by this, and previous studies may also be reproductively isolated.

For most of the last 250,000 years, southern New Guinea and northern Australia were connected by land bridges due to lower sea levels. In particular, what is now northern Queensland was connected to southern New Guinea for more than 90 per cent of the last 250,000 years, while the Northern Territory was only connected to New Guinea for <10 per cent of this period (Voris, 2000). Following the most recent glacial maximum, the Northern Territory separated from New Guinea approximately 12,000 years bp and Queensland separated from southern new Guinea as recently as 7000 years bp (Lambeck & Nakada, 1990; Nix & Kalma, 1975). Close relationships in the mtDNA haplotype network reflect these recent connections, and it is likely that the populations from Queensland and southern New Guinea formed a large metapopulation encompassing this area during the Pleistocene. Nuclear microsatellites support this hypothesis, as the Queensland and southern New Guinean populations are closely related for these markers.

The Northern Territory population is the most genetically distant of any population in microsatellite analyses as observed through the pairwise fixation indices. This may be explained by the reduced period of time that the Northern Territory was connected to New Guinea during the Pleistocene. Although there were land bridges connecting the Northern Territory to New Guinea relatively recently, the climate during glacial maxima in large parts of Australia was much drier than it is today (Williams et al., 2009). This means that that there would have been little opportunity for connectivity between the Northern Territory and other populations in Queensland and New Guinea, even when New Guinea was connected to the Northern Territory directly. Today *An*. *hinesorum* in the Northern Territory could be a remnant population with a restricted distribution (Cooper et al., 2002). Additionally, the monsoonal climate in the Northern Territory drives intense dry periods, likely causing this population to go through regular bottlenecks, allowing greater potential for genetic drift to occur. These climatic and biogeographic factors working on a small, isolated population may explain why the Northern Territory population appears so distinct for the microsatellite markers used in this study.

### 4.2 Evolution of exclusive zoophagy in the Solomon Archipelago

The *Anopheles farauti* complex shows variation in human host preference. Zoophagy is a derived trait in this complex that has evolved at least twice in the Solomon Archipelago: once in *An*. *irenicus* (another exclusively zoophagic species in the *An*. *farauti* group) and at least once in *An*. *hinesorum* (Ambrose et al., 2012; Beebe et al., 2000; Foley et al., 1994). This group therefore provides a useful system to study the genetic basis of human host preference in mosquitoes. Specialization in this group has occurred in the opposite direction to that in *An*. *gambiae* s.s. and *Ae*. *aegypt*i, with species in the *An*. *farauti* complex having evolved from anthropophagic generalists to exclusively zoophagic species (and populations; Ambrose et al., 2012). The *An*. *farauti* complex therefore provides a useful counterpoint for comparison to other well‐studied mosquito systems.

Human landing catches performed during studies in Bougainville and Guadalcanal have failed to collect *An*. *hinesorum* despite productive larval habitats near the human landing catches (Cooper & Frances, 2002; Foley et al., 1994). Thus, the zoophagic trait appears to be fixed in *An*. *hinesorum* populations from the northern and southern Solomon Archipelago. The hosts that these populations are feeding upon remain unknown but there was probably a limited range of hosts available on the Solomon Archipelago at the time of initial colonization, and sizeable mammals may not have been present (Ambrose et al., 2012). *Anopheles hinesorum* in Australia and New Guinea are attracted to carbon dioxide baited traps while populations in the Solomon Archipelago are not (Cooper & Frances, 2002; Cooper et al., 2009; Foley et al., 1994; Van Den Hurk et al., 1997), a phenotypic difference indicating that the colonization of these islands may have driven the adaptation of *An*. *hinesorum* to ectothermic hosts. Other animals, including insects, have experienced host shifts and specialization when colonizing islands (Jorge et al., 2018; Simberloff, 1974; Tseng et al., 2018; Yassin et al., 2016), and the Solomon Archipelago supports an abundant and diverse frog and reptile fauna (Morrison et al., 2007; Pikacha et al., 2016), providing an plentiful potential food source.

Mitochondrial DNA data starkly show that there are two distinct lineages of *An*. *hinesorum* occupying the north and south of the Solomon Archipelago. These most likely reflect two distinct migrations of the species to the islands from New Guinea or elsewhere: firstly, an old migration whose haplotypes dominate the northern islands, likely dispersing from New Guinea through the New Britain archipelago and then a more recent migration into the southern islands, possibly from the Papuan peninsula. The five samples from Nggela as well as most samples from Santa Isabel belong to the northern haplotype group, likely indicative of historical Pleistocene connectivity through the north‐eastern islands of the archipelago—Bougainville, Santa Isabel and the Nggela Islands.

Three distinct evolutionary scenarios may have generated the observed pattern of exclusive zoophagy in two divergent mtDNA lineages of *An*. *hinesorum* found on the Solomon Archipelago. These include the convergent evolution of the trait, the evolution of the trait in one population and subsequent transmission via gene flow to the other, and the replacement of mitochondria (via selective sweep) in a widespread zoophagic population in the Solomon Archipelago. Despite the southern and northern Solomon Archipelago mtDNA lineages being highly divergent, all populations from the Archipelago form a closely related group at the nuclear DNA level. These close relationships suggest that recent nuclear gene flow has occurred between them or that they share a more recent common ancestor than their mtDNA genomes suggest. The northern mtDNA lineage dominates the islands extending from Bougainville in the north to Nggela in the south, although several individuals sampled from Santa Isabel possess a haplotype commonly found on Guadalcanal (southern lineage). This pattern suggests either past connectivity between these islands or long‐distance migration of females.

Genetic connectivity between islands in the Solomon Archipelago is also indicated by the microsatellite data. STRUCTURE plots and DAPC *compoplots* show mixed assignment of individuals from different islands in the Solomon Archipelago. Comparatively low fixation indices also indicate that recent gene flow has occurred. This result makes sense given that most islands of the Solomon Archipelago were connected by land bridges and formed a larger island known as Greater Bukida, separated from Guadalcanal by only 2 km of ocean at times of lowest sea level (Mayr & Diamond, 2001). The Western Province islands were still isolated during this period. Interestingly, individuals sampled from the Western Province possessed mtDNA haplotypes from both the highly divergent northern and southern lineages and the eastern peninsula of New Guinea.

It is likely that microsatellite differentiation between island populations reflects postglacial divergence. Previously we hypothesized that zoophagy may have evolved independently in the two divergent island mitochondrial lineages of *An*. *hinesorum* due to selection on new migrants. However, it now seems likely that zoophagy evolved in one lineage and moved through the Archipelago by migration and gene flow. As we do not yet know the genetic basis of the trait, we cannot be sure which scenario has resulted in the evolution and spread of zoophagy in these islands.

### 4.3 Emergence of anthropophagy in the Solomon Archipelago: Recent gene flow from New Guinea and implications for malaria transmission

Recent fieldwork in Solomon Islands Western Province identified *An*. *hinesorum* feeding on humans (Burkot et al., 2018). This is an important finding as the species has rarely been collected feeding on humans in the Solomon Archipelago during many previous attempts to collect *An*. *hinesorum* in human landing catches, despite abundant larvae in the immediate landscape. The mtDNA (*COI*) of samples from this population fell into three genetically distinct and geographically defined groups: one from New Guinea (Papuan Peninsula) and two from the Solomon Archipelago (northern and southern lineages). One Western Province haplotype sampled in eleven individuals is identical to a sequence sampled from an anthropophagic population in New Guinea—the only case of haplotype sharing observed between Solomon Archipelago and New Guinean populations. This raises the possibility that female *An*. *hinesorum* from New Guinea were able to arrive in the Western Province of the Solomon Islands, reproduce with local mosquitoes, and in the process transfer anthropophagy to this island population.

There are two obvious recent dispersal routes for *An*. *hinesorum* from New Guinea to the Western Province. During the World War II Pacific engagement, American airbases connected the Western Province to New Guinea via airstrips in Lae and Milne Bay (the eastern point of the Papuan peninsula—see Figure S3). At this time, anthropophagic *An*. *hinesorum* were abundant at these New Guinean airbase sites (Beebe & Cooper, 2002) and there would have been ample opportunity for human‐seeking female mosquitoes to enter aircraft destined for the Munda airstrip in Western Province. These females could have arrived in Munda—where plentiful larval sites and human hosts were available—within a day. There are also regular contemporary shipping movements between Lae (northern Papuan Peninsula) and Munda (Western Province Solomon Islands) which may provide additional ongoing human‐aided dispersal opportunities.

Even though a commonly sampled mitochondrial haplotype in the Solomon Islands Western Province is identical to a haplotype sampled in a New Guinean population—the only case of haplotype sharing between New Guinean and Solomon Archipelago populations—the nuclear microsatellites suggest the genomes of the Western Province population appear to be mostly of native Solomon Archipelago origin. This is shown by the close relationships at microsatellite loci between individuals from Western Province and the rest of the Solomon Archipelago. In mosquitoes, olfaction is the primary sense that governs host preference (Takken, 1991), and it is likely that only small regions (e.g. a small number of olfactory genes) of the genome are associated with the ability to detect and feed on humans (Raji & DeGennaro, 2017). Thus, it is possible that small parts of the New Guinean nuclear genome (that are associated with anthropophagy and likely olfactory in function) have been retained in the anthropophagic Solomon Islands Western Province population. For this reason, the Western Province Solomon Island population may be central to identifying genes or genomic regions associated with mosquito anthropophagy in future comparative population genomics investigations.

Today, the only common species known to transmit malaria in the Solomon Archipelago is the coastally restricted *An*. *farauti* (Beebe et al., 2015). The emergence of anthropophagy in a population of *An*. *hinesorum* from the Solomon Archipelago has serious implications for the transmission of malaria, especially if this phenotype spreads through other populations in the Archipelago. *Anopheles hinesorum* is common and abundant through the Solomon Islands showing larval site plasticity, and existing both inland and at elevation (Beebe et al., 2015). If anthropophagy provides a selective advantage (i.e. blood source availability and improved fecundity), it may spread quickly resulting in the emergence of a second common malaria vector in the Solomon Archipelago. This could have serious implications for the spread of malaria in the Solomon Archipelago due to the high abundance of *An*. *hinesorum* through the Solomon Islands.

## 5 CONCLUSIONS

In this study, we have achieved a more complete understanding of population genetic relationships of *An*. *hinesorum* in the Western Pacific, clarifying population subdivisions. This lays the groundwork necessary to use this species as a novel model system for studying human host preference in mosquitoes. Large mtDNA divergences likely do not indicate species boundaries, as nuclear gene flow is evident between some highly diverged lineages in the Solomon Archipelago. Although we cannot be certain that exclusive zoophagy in the Solomon Island populations was transmitted between these divergent lineages via gene flow, our results suggest that gene flow between islands of the Archipelago has occurred. Further work is necessary to disentangle the hypotheses regarding the origins of zoophagy in the Solomon Archipelago. We detected New Guinean mitotypes in a recently discovered anthropophagic population from the Solomon Islands indicating that human‐mediated transport of the species may have resulted in anthropophagy being introduced to the Archipelago. The emergence of this phenotype may have ramifications for the epidemiology and transmission of malaria on the Solomon Archipelago; specifically, it may result in increased malaria transmission in inland villages.

## CONFLICT OF INTEREST

The authors declare no conflicts of interest.

## Supporting information

Fig S1‐S3

Table S1‐S4

Supplementary Material

## Data Availability

The data that support the findings of this study are openly available in Dryad at http://doi.org/10.5061/dryad.bg79cnpb8
