# Supplementary material for: Gene flow between island populations of the malaria mosquito, Anopheles hinesorum, may have contributed to the spread of divergent host preference phenotypes
Source: Evol Appl. 2021 Aug 23;14(9):2244–57. doi: 10.1111/eva.13288 (PMC8477600; doi:10.1111/eva.13288)
Supplement: Supplementary file 1 — Fig S1‐S3 [file EVA-14-2244-s002.docx]

**Supplementary Figure 1: Multivariate scatter plots from PCA and t-SNE analyses.**


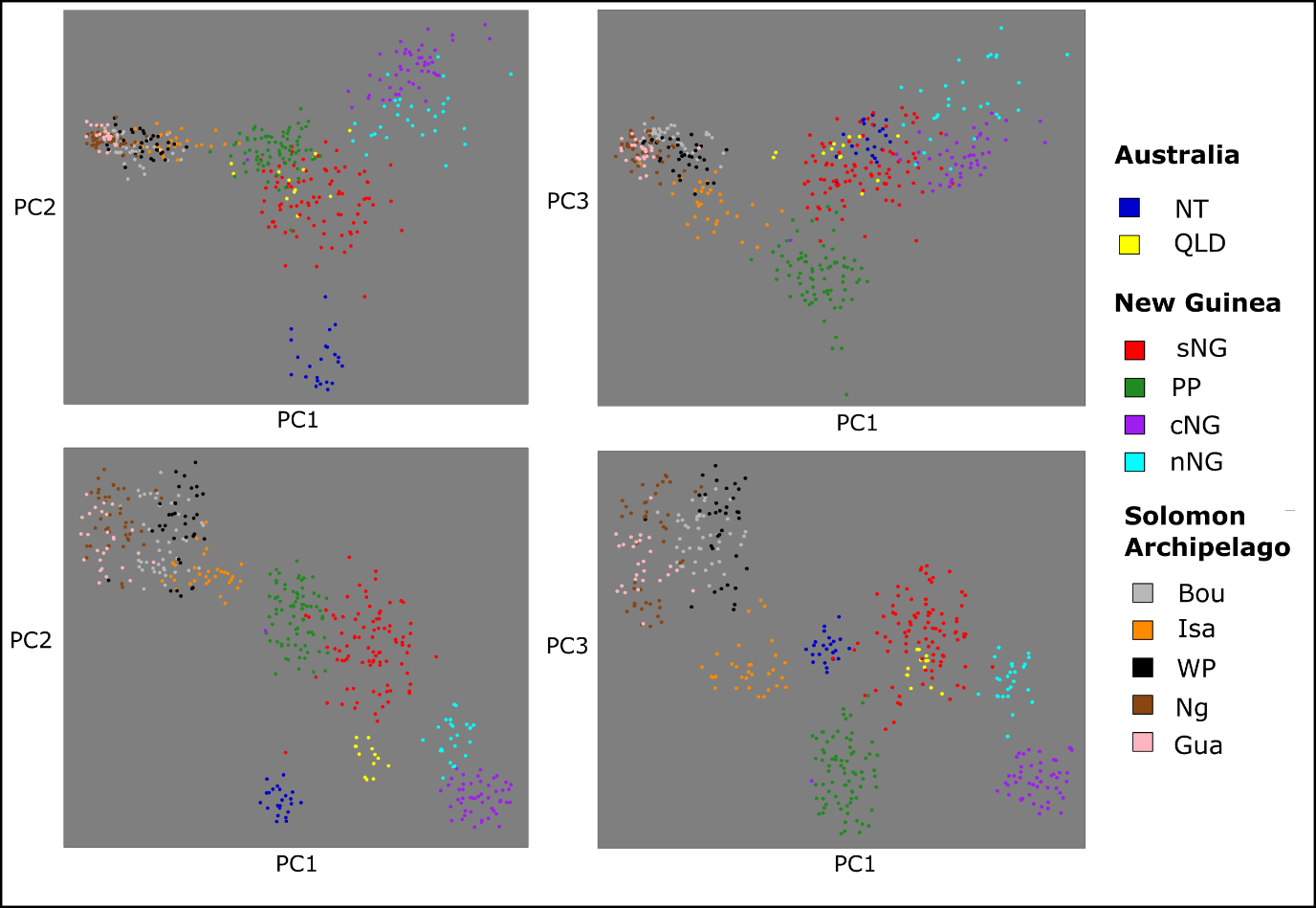


**Supplementary Figure 1: Multivariate analyses of *An. hinesorum* (14 nuclear microsatellite loci).**

**Top Panels:** Scatter plots of first three principal components from principal component analysis (PCA).

**Bottom Panels:** Scatter plots of first three principal components from t-SNE (t-distributed stochastic neighbour embedding).

**Supplementary Figure 2:** **Discriminant analysis of principal components (DAPC) for Solomon Archipelago populations of *An. hinesorum*.**


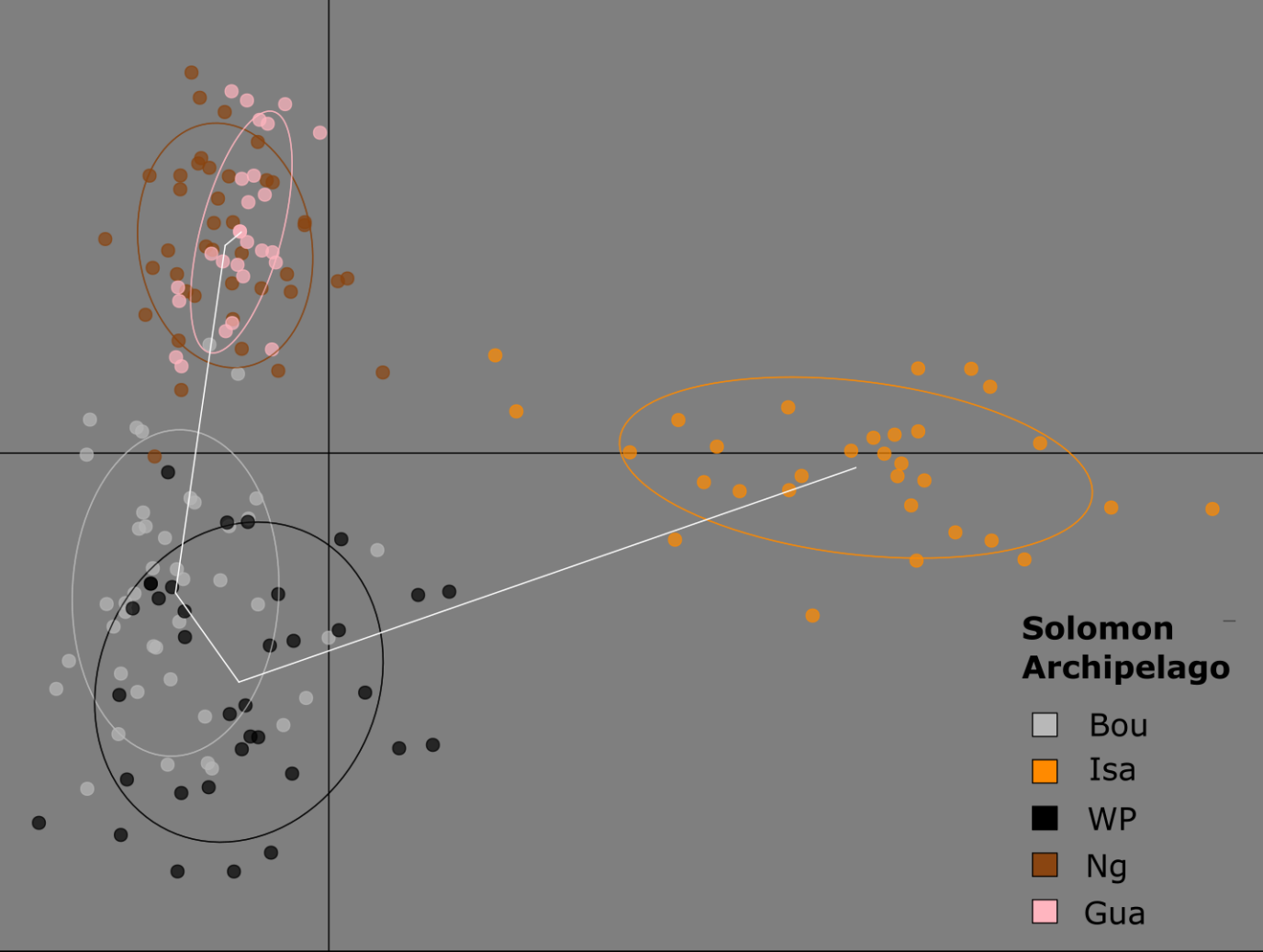


**Supplementary Figure 2: Discriminant analysis of principal components (DAPC) for Solomon Archipelago populations of *An. hinesorum*.** The above figure is a scatter plot of a DAPC generated from *An. hinesorum* microsatellite data from Solomon Archipelago populations. Each point represents a single individual and distance between points is negatively correlated with how closely related they are. Individuals are coloured by population as shown in the key above. The white line connecting groups is an estimated minimum spanning tree.

**Supplementary Figure 3:** **Map of locations referred to in the manuscript.**

**
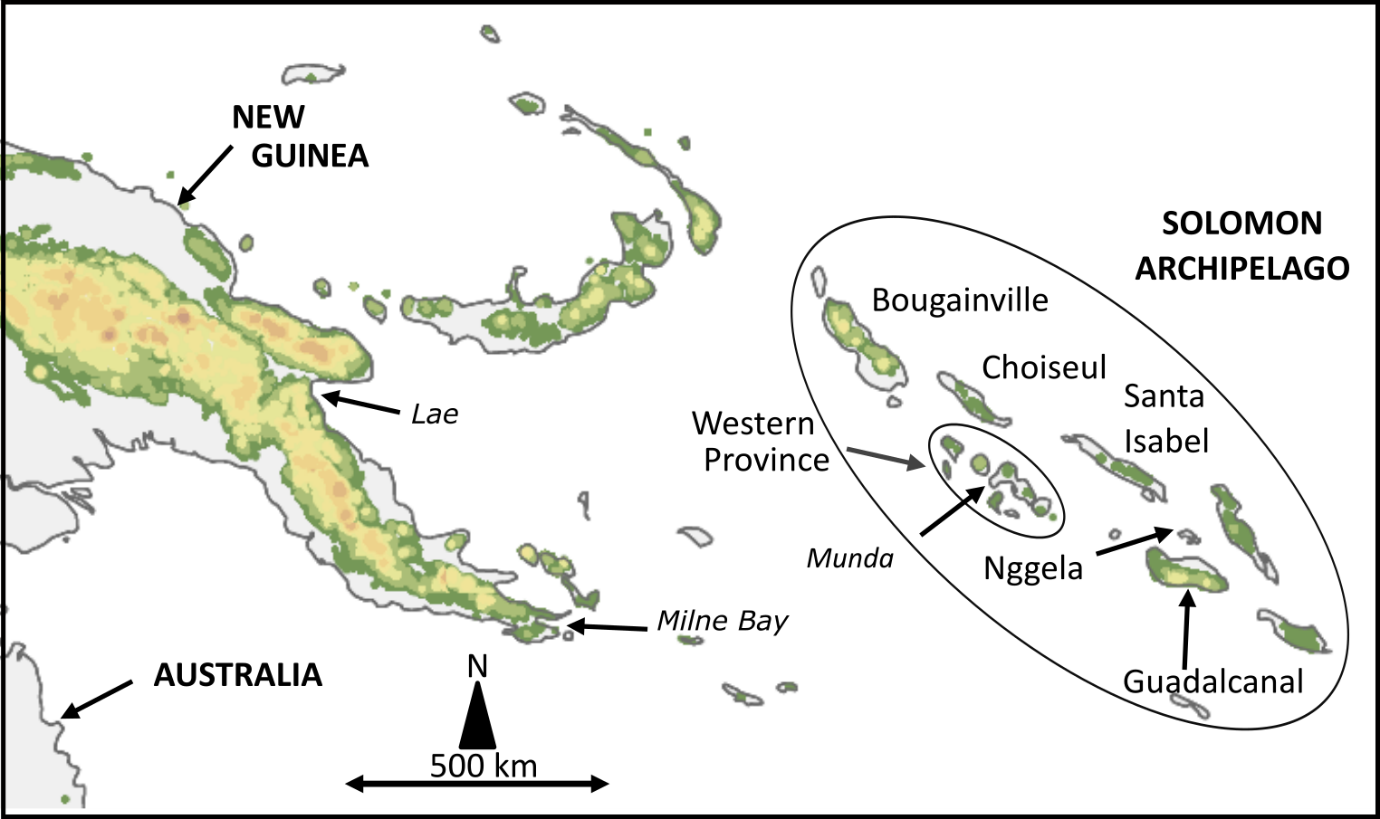
**

**Supplementary Figure 3: Map of locations referred to in the manuscript.** The above figure shows additional locations referred to in the Discussion section of the manuscript.
