# Supplementary material for: Gene flow between island populations of the malaria mosquito, Anopheles hinesorum, may have contributed to the spread of divergent host preference phenotypes
Source: Evol Appl. 2021 Aug 23;14(9):2244–57. doi: 10.1111/eva.13288 (PMC8477600; doi:10.1111/eva.13288)
Supplement: Supplementary file 2 — Table S1‐S4 [file EVA-14-2244-s003.docx]

| Primer name | Forward primer | Reverse primer | Dye | Length range (bp) | N alleles | HWE | Nulls |
| --- | --- | --- | --- | --- | --- | --- | --- |
| *hin-di2* | ACGAGACGACCGTTCAAATG | GGGAGGGCTGTGGTTTAGAA | FAM | 175 - 199 | 12 | - | sNG |
| *hin-di6* | AAGTCCGATCGAGCGTAGTC | CAAAGTGCAACCGAGGAGTC | NED | 158 - 186 | 11 | - | PP, sNG |
| *hin-di8* | GTTCGATAAGGGAAAGCGGC | CATTTGACGACGACACCCTG | PET | 333 - 353 | 11 | Bou | sNG |
| *hin-di12* | TCGGCTGGGAGGATTACATG | GGCGTCGTCACTCATCAATC | VIC | 138 - 174 | 12 | sNG, Ng, Gua | Isa, Ng, Gua, PP,sNG, WPSI |
| *hin-di14* | TCGCCTAGACAATCATCGGG | CCGTAACAGGGATTGCAAGG | NED | 271 - 343 | 27 | - | NT, PP QLD, sNG |
| *hin-tri1* | GAAACTTTGCCCTGCTGCC | CGTCCAGGTGTCTAGGTGAA | NED | 207 - 255 | 14 | NT, nNG | NT, PP, QLD, sNG, nNG, WPSI |
| *hin-tri3* | GAAACACACGGCCCTTGTAC | GCATGCAAGCAGTTGTAGCA | PET | 307 - 352 | 13 | cNG | cNG, nNG |
| *hin-tri4* | GCGGACAAATTTGCAACCAC | CATATGCGCGAGAGAGGAGT | FAM | 260 - 311 | 15 | - | cNG, PP, sNG |
| *hin-tri6* | GCTGATAGTCGAGCCACAAG | GTCTTCACCATCGGCACTCT | NED | 201 - 255 | 10 | Isa, WPSI, sNG | Isa, sNG, WPSI |
| *hin-tri7* | TATTACTATTCCCGGGCGCG | GATCGATCGGTGGATGTGTT | VIC | 139 - 163 | 9 | - | - |
| *hin-tri10* | CTCGCGCAGGATTCGATTAC | GAGATCTGGTCGCTGCTACA | VIC | 341 - 359 | 8 | - | PP, sNG |
| *hin-tri12* | CCACAAACCTTCCGATAGCC | CGTTCAGGACATTGTTCCGG | FAM | 227 - 266 | 13 | sNG, cNG | cNG, sNG |
| *hin-tri19* | TCTTGCGAACCCTCCTTCTC | CGCGTGGTAAATTGGGTAGG | PET | 117 - 174 | 12 | Isa, sNG, cNG, Bou | Isa, Bou, cNG, QLD, sNG, nNG, WPSI |
| *hin-tri24* | TGCTCAAGTTGCTGATCACC | CTGGACGCAATGATCGCTTT | PET | 169 - 190 | 8 | - | sNG |

**Supplementary Table 1: Microsatellite primer and locus information.** All forward primers also included an M13 tail at their 5’ end, designed to bind to the M13 labelled dyes used. HWE = populations that show evidence of deviating from Hardy-Weinberg; Nulls = populations that may have null alleles.

|  | NT | QLD | sNG | pp | cNG | nNG | nSI | Isa | WPSI | Gua | Ng |
| --- | --- | --- | --- | --- | --- | --- | --- | --- | --- | --- | --- |
| NT | 0.000 |  |  |  |  |  |  |  |  |  |  |
| QLD | 0.219 | 0.000 |  |  |  |  |  |  |  |  |  |
| sNG | 0.164 | 0.099 | 0.000 |  |  |  |  |  |  |  |  |
| pp | 0.228 | 0.152 | 0.089 | 0.000 |  |  |  |  |  |  |  |
| cNG | 0.275 | 0.190 | 0.134 | 0.170 | 0.000 |  |  |  |  |  |  |
| nNG | 0.228 | 0.138 | 0.088 | 0.157 | 0.117 | 0.000 |  |  |  |  |  |
| nSI | 0.374 | 0.262 | 0.197 | 0.251 | 0.360 | 0.288 | 0.000 |  |  |  |  |
| Isa | 0.315 | 0.216 | 0.145 | 0.121 | 0.276 | 0.242 | 0.198 | 0.000 |  |  |  |
| WPSI | 0.342 | 0.243 | 0.175 | 0.206 | 0.323 | 0.250 | 0.049 | 0.148 | 0.000 |  |  |
| Gua | 0.402 | 0.306 | 0.226 | 0.264 | 0.385 | 0.310 | 0.084 | 0.188 | 0.084 | 0.000 |  |
| Ng | 0.417 | 0.337 | 0.247 | 0.278 | 0.396 | 0.324 | 0.115 | 0.199 | 0.134 | 0.069 | 0.000 |

**Supplementary Table 2: Pairwise G^ST^ between *An. hinesorum* populations based on 14 microsatellite loci.** NT = Northern Territory Australia; Qld = Queensland Australia; sPNG = southern Papua New Guinea; cPNG = central Papua New Guinea; PP = Papuan Peninsula New Guinea; nPNG = northern New Guinea; Bou = Bougainville and Buka Solomon Islands; Isa = Santa Isabel Solomon Islands; WPSI = Western Province Solomon Islands; Gua = Guadalcanal Solomon Islands; Ng = Nggela Solomon Islands.

### Supplementary Table 3: Pairwise fixation indices (G’ST) between An. hinesorum populations.

|  | **NT** | **QLD** | **sNG** | **pp** | **cNG** | **nNG** | **nSI** | **Isa** | **WPSI** | **Gua** | **Ng** |
| --- | --- | --- | --- | --- | --- | --- | --- | --- | --- | --- | --- |
| **NT** | 0.000 |  |  |  |  |  |  |  |  |  |  |
| **QLD** | 0.562 | 0.000 |  |  |  |  |  |  |  |  |  |
| **sNG** | 0.580 | 0.357 | 0.000 |  |  |  |  |  |  |  |  |
| **pp** | 0.660 | 0.444 | 0.369 | 0.000 |  |  |  |  |  |  |  |
| **cNG** | 0.786 | 0.548 | 0.542 | 0.556 | 0.000 |  |  |  |  |  |  |
| **nNG** | 0.792 | 0.485 | 0.453 | 0.635 | 0.463 | 0.000 |  |  |  |  |  |
| **nSI** | 0.766 | 0.539 | 0.545 | 0.581 | 0.821 | 0.780 | 0.000 |  |  |  |  |
| **Isa** | 0.741 | 0.513 | 0.470 | 0.325 | 0.726 | 0.765 | 0.378 | 0.000 |  |  |  |
| **WPSI** | 0.754 | 0.539 | 0.526 | 0.514 | 0.793 | 0.736 | 0.089 | 0.303 | 0.000 |  |  |
| **Gua** | 0.806 | 0.617 | 0.609 | 0.597 | 0.856 | 0.819 | 0.138 | 0.351 | 0.148 | 0.000 |  |
| **Ng** | 0.822 | 0.670 | 0.656 | 0.620 | 0.870 | 0.843 | 0.187 | 0.367 | 0.233 | 0.109 | 0.000 |

**Table 2: Pairwise fixation indices (G’ST) between An. hinesorum populations based on 14 microsatellite loci.**

|  | NT | QLD | sNG | pp | cNG | nNG | nSI | Isa | WPSI | Gua | Ng |
| --- | --- | --- | --- | --- | --- | --- | --- | --- | --- | --- | --- |
| NT | 0.000 |  |  |  |  |  |  |  |  |  |  |
| QLD | 0.439 | 0.000 |  |  |  |  |  |  |  |  |  |
| sNG | 0.498 | 0.286 | 0.000 |  |  |  |  |  |  |  |  |
| pp | 0.560 | 0.345 | 0.307 | 0.000 |  |  |  |  |  |  |  |
| cNG | 0.704 | 0.442 | 0.472 | 0.465 | 0.000 |  |  |  |  |  |  |
| nNG | 0.730 | 0.403 | 0.401 | 0.567 | 0.392 | 0.000 |  |  |  |  |  |
| nSI | 0.627 | 0.376 | 0.434 | 0.441 | 0.721 | 0.691 | 0.000 |  |  |  |  |
| Isa | 0.621 | 0.378 | 0.380 | 0.231 | 0.621 | 0.690 | 0.225 | 0.000 |  |  |  |
| WPSI | 0.626 | 0.392 | 0.426 | 0.388 | 0.695 | 0.648 | 0.041 | 0.182 | 0.000 |  |  |
| Gua | 0.675 | 0.448 | 0.495 | 0.452 | 0.766 | 0.737 | 0.059 | 0.200 | 0.070 | 0.000 |  |
| Ng | 0.695 | 0.502 | 0.543 | 0.473 | 0.784 | 0.767 | 0.081 | 0.209 | 0.114 | 0.043 | 0.000 |

**Supplementary Table 4: Pairwise DEST between *An. hinesorum* populations based on 14 microsatellite loci.** NT = Northern Territory Australia; Qld = Queensland Australia; sPNG = southern Papua New Guinea; cPNG = central Papua New Guinea; PP = Papuan Peninsula New Guinea; nPNG = northern New Guinea; Bou = Bougainville and Buka Solomon Islands; Isa = Santa Isabel Solomon Islands; WPSI = Western Province Solomon Islands; Gua = Guadalcanal Solomon Islands; Ng = Nggela Solomon Islands.
