# Supplementary material for: Gene flow between island populations of the malaria mosquito, Anopheles hinesorum, may have contributed to the spread of divergent host preference phenotypes
Source: Evol Appl. 2021 Aug 23;14(9):2244–57. doi: 10.1111/eva.13288 (PMC8477600; doi:10.1111/eva.13288)
Supplement: Supplementary file 3 — Supplementary Material [file EVA-14-2244-s001.pdf]

CLUMPAK main pipeline - Job 1558131284 summary

Major modes for the uploaded data:

K=2

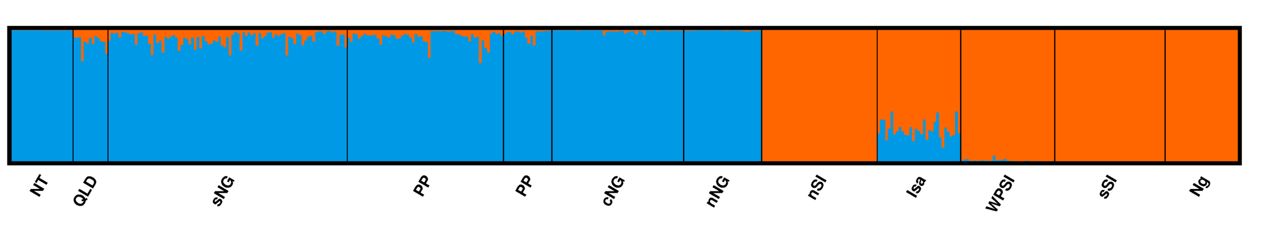

K=3

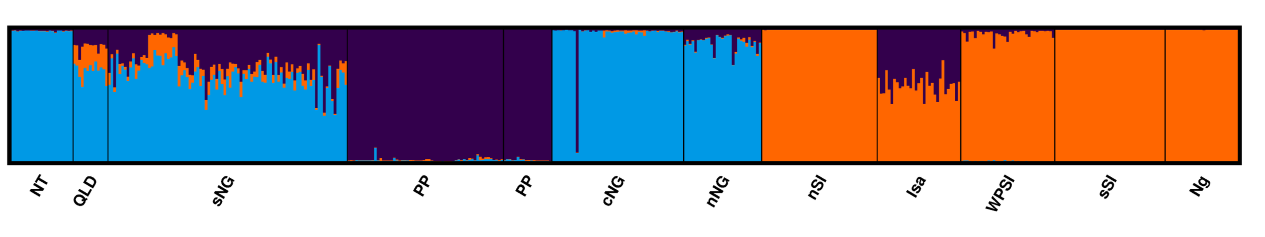

K=4

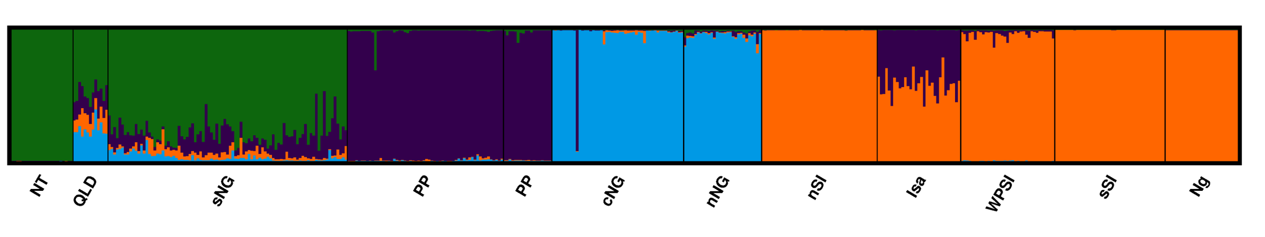

K=5

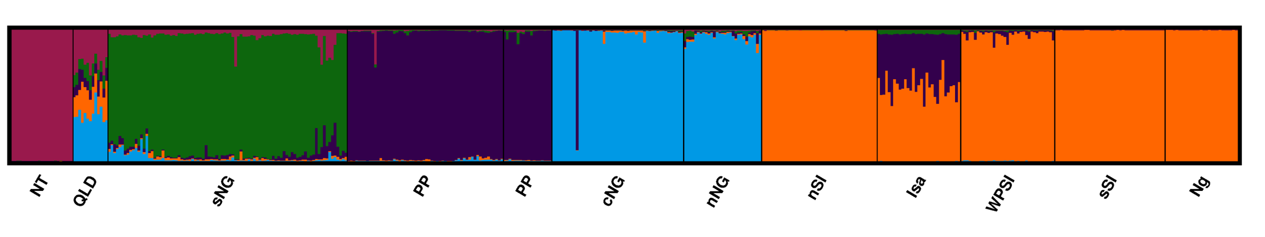

K=6

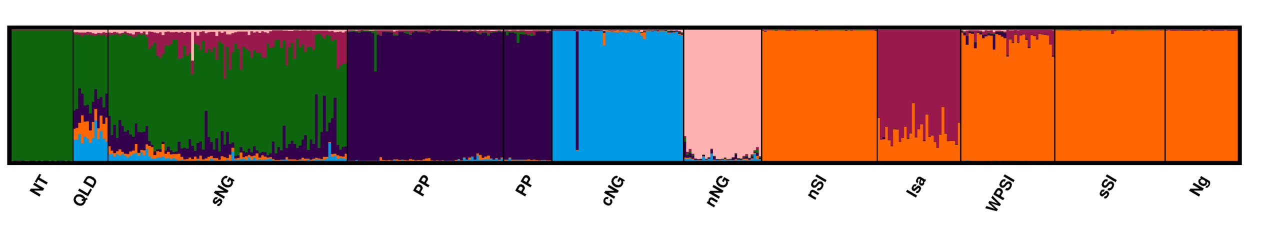

K=7

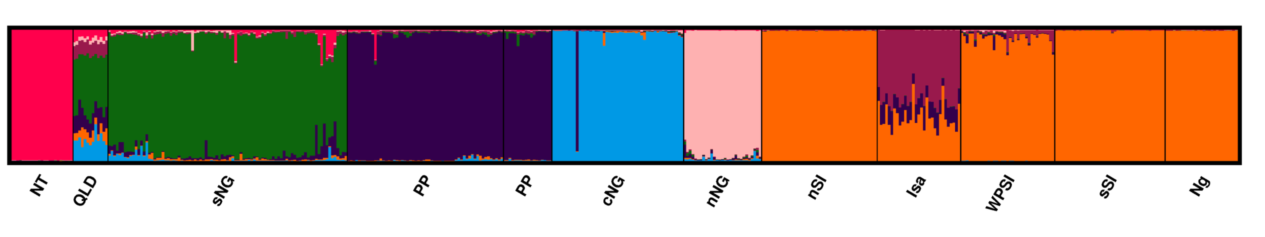

K=8

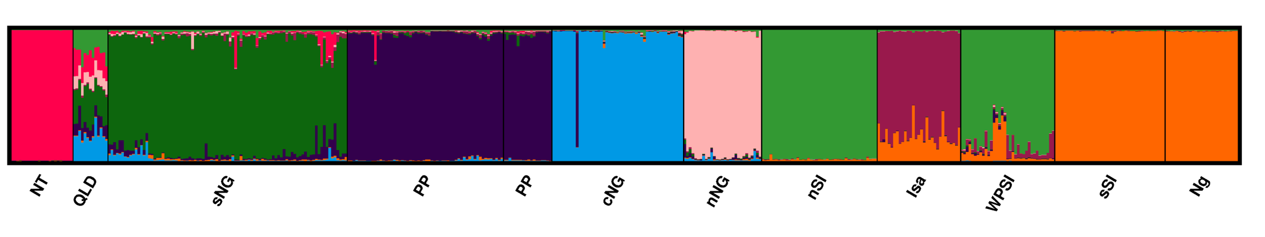

K=9

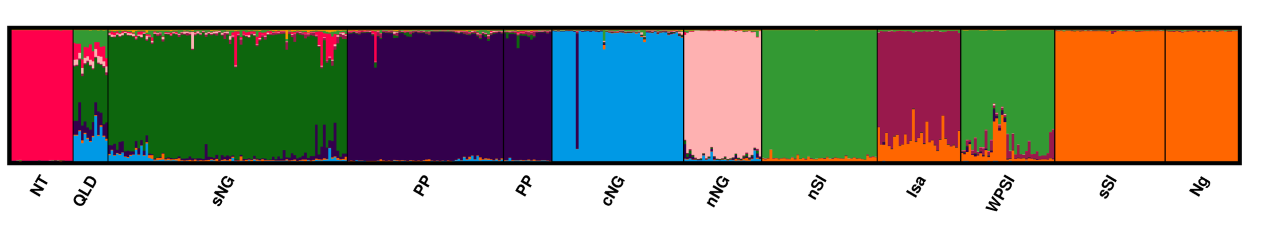

K=10

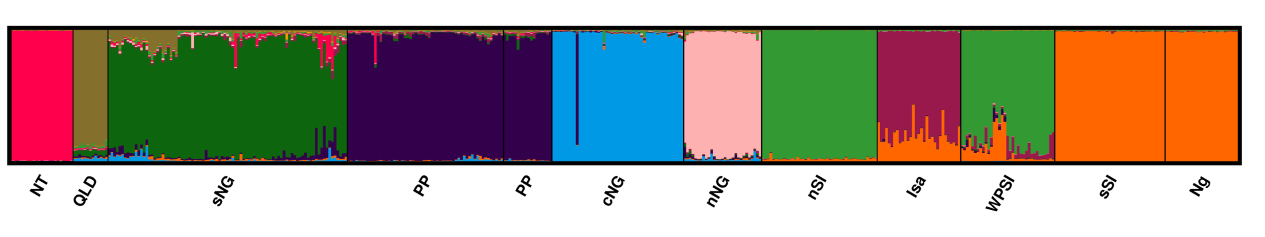

K=11

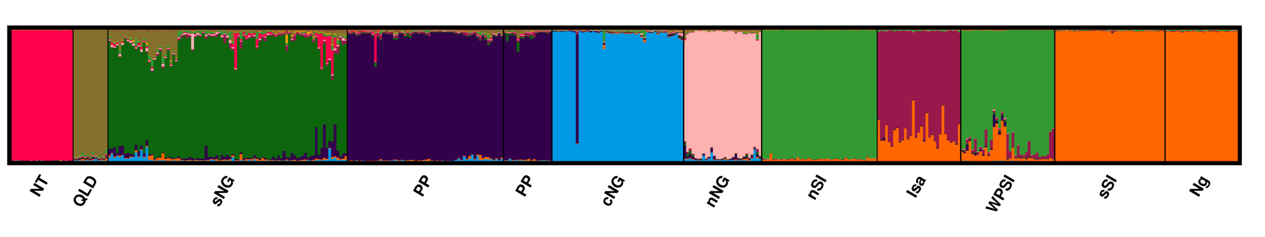

K=12

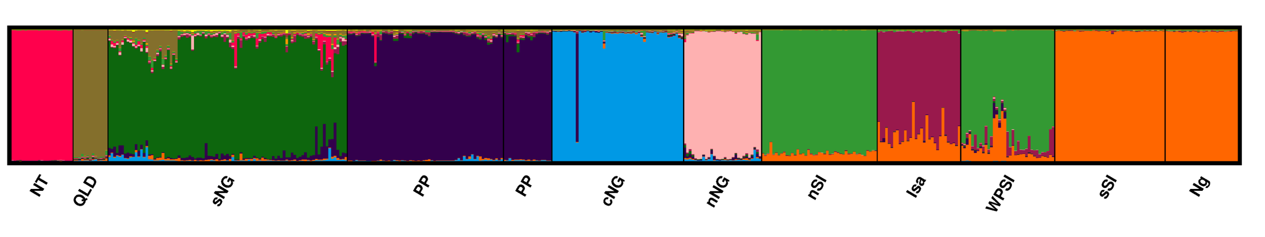

K=13

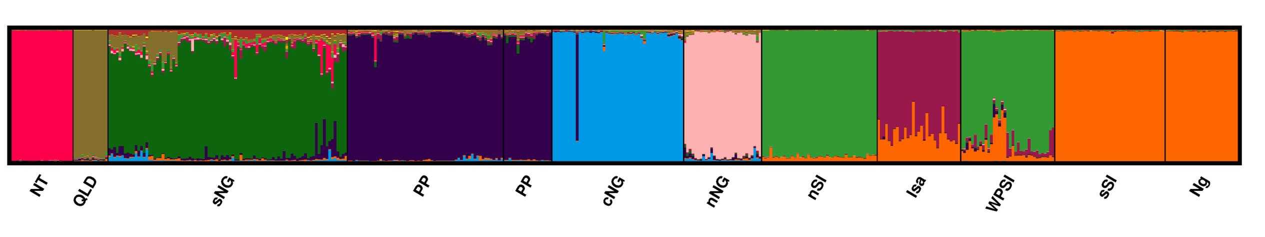

K=14

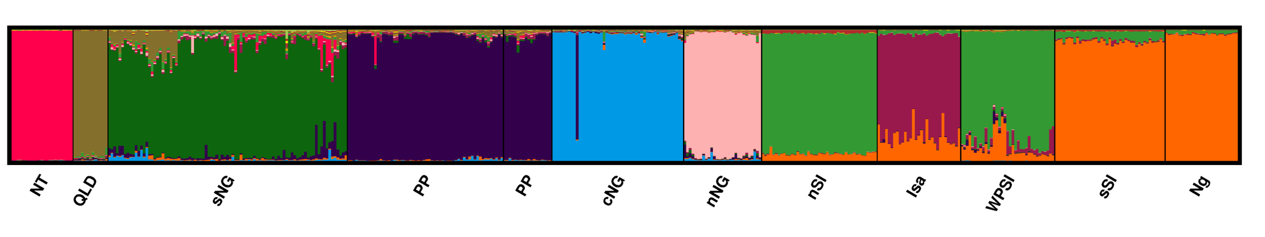

K=15

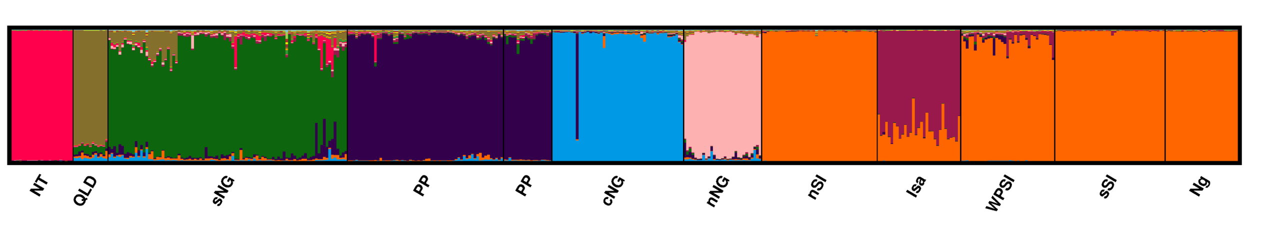

Minor modes for the uploaded data:

K=3 MinorCluster1

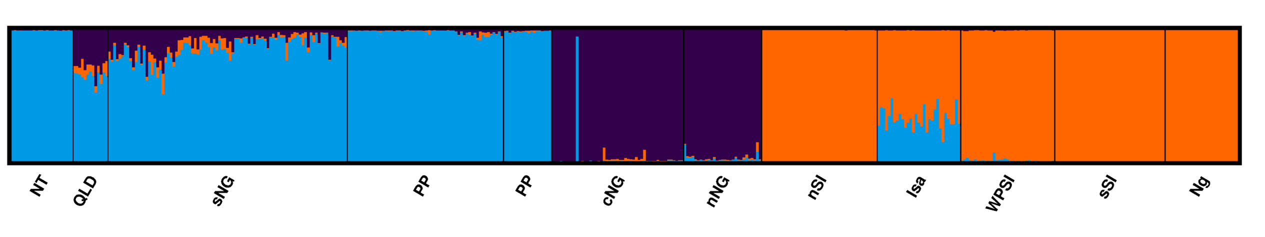

K=3 MinorCluster2

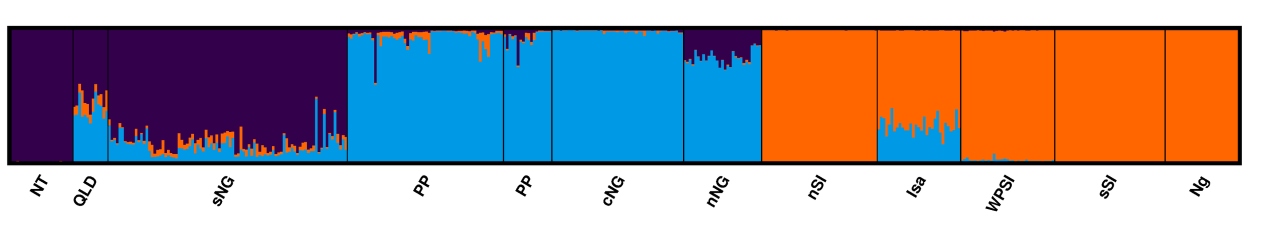

K=5 MinorCluster1

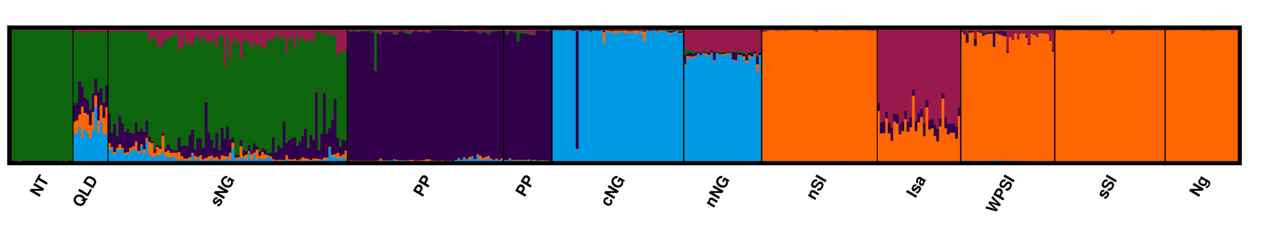

K=6 MinorCluster1

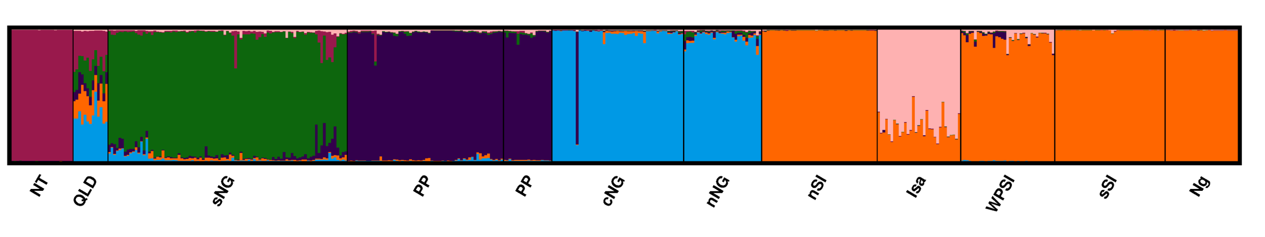

K=6 MinorCluster2

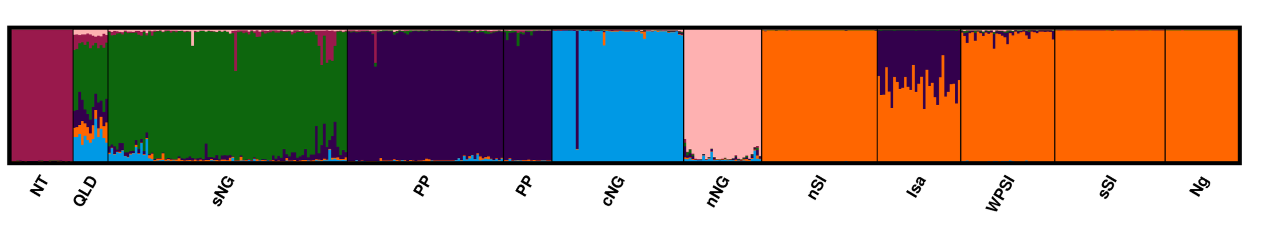

K=6 MinorCluster3

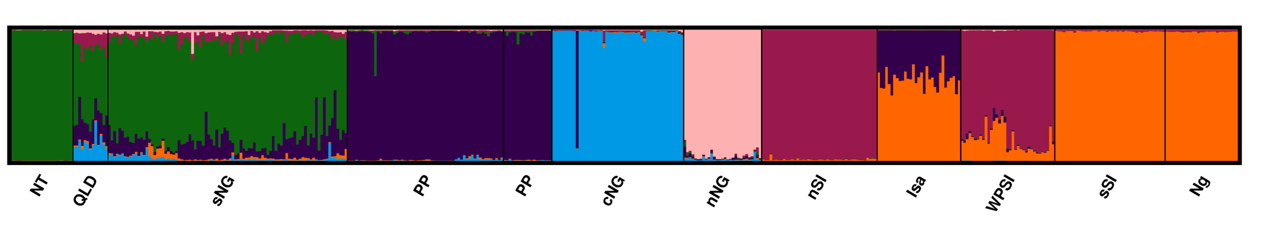

K=6 MinorCluster4

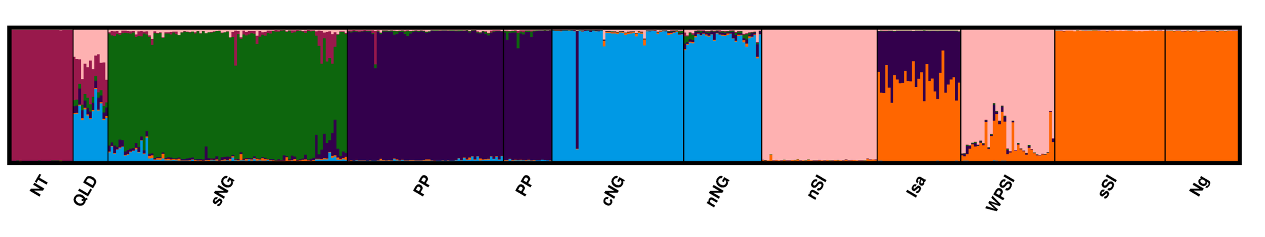

K=7 MinorCluster1

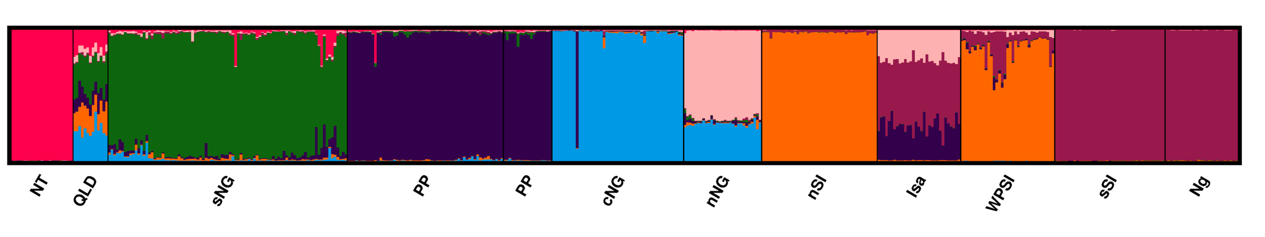

K=8 MinorCluster1

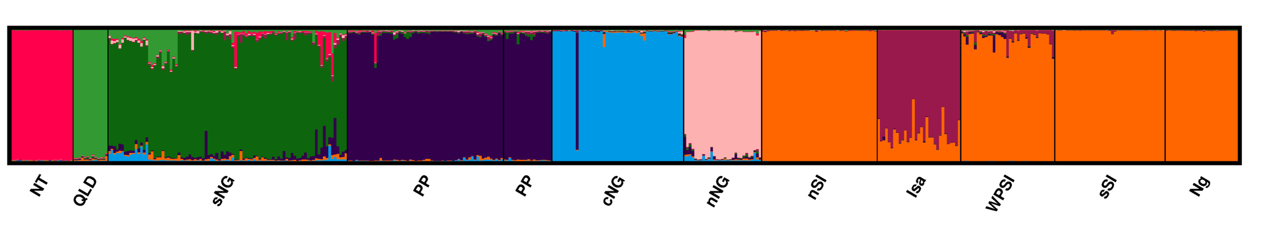

K=8 MinorCluster2

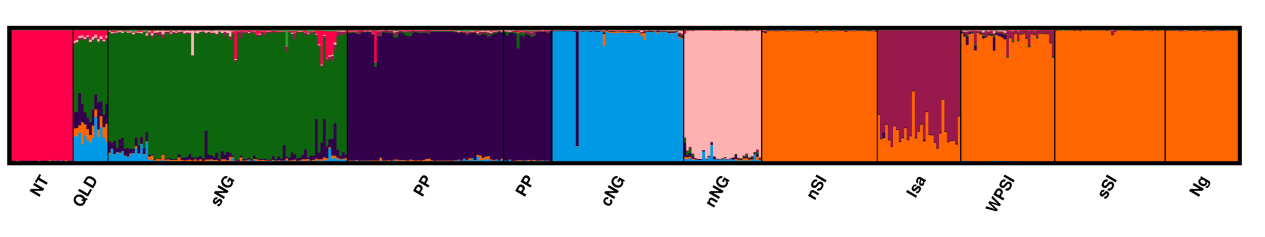

K=9 MinorCluster1

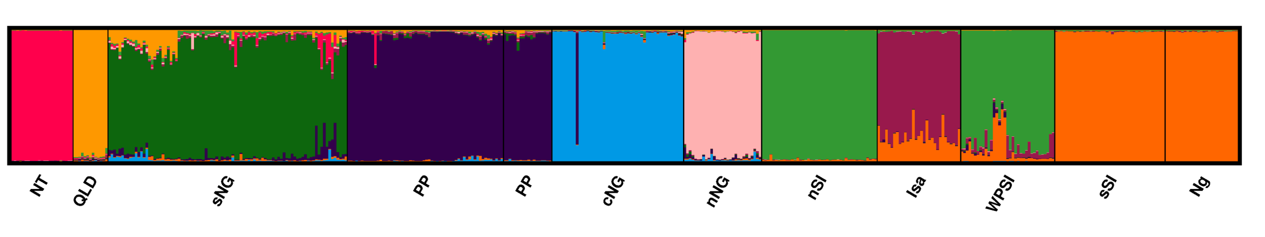

K=9 MinorCluster2

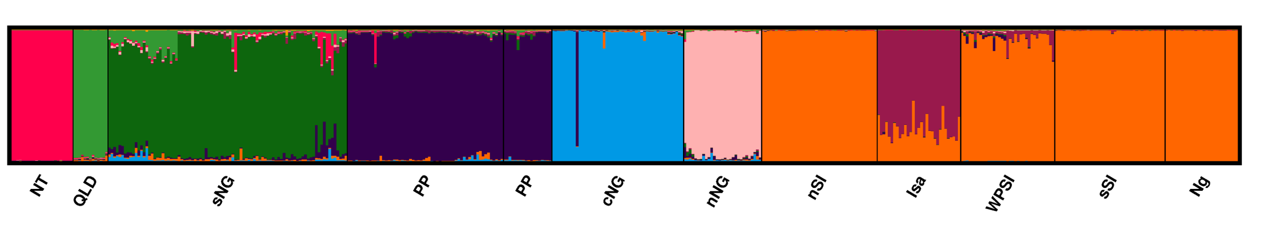

K=9 MinorCluster3

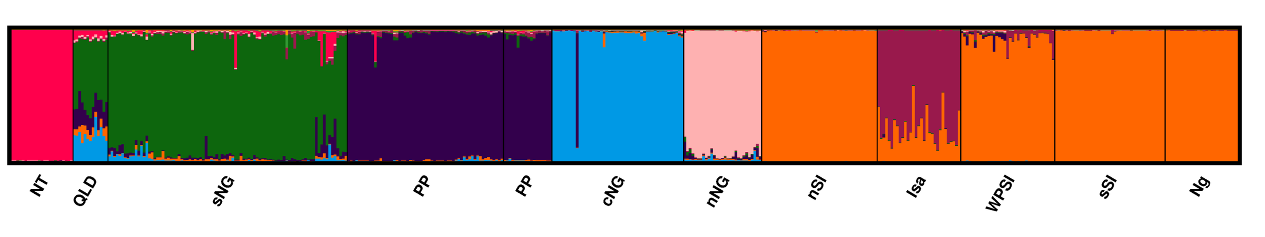

K=10 MinorCluster1

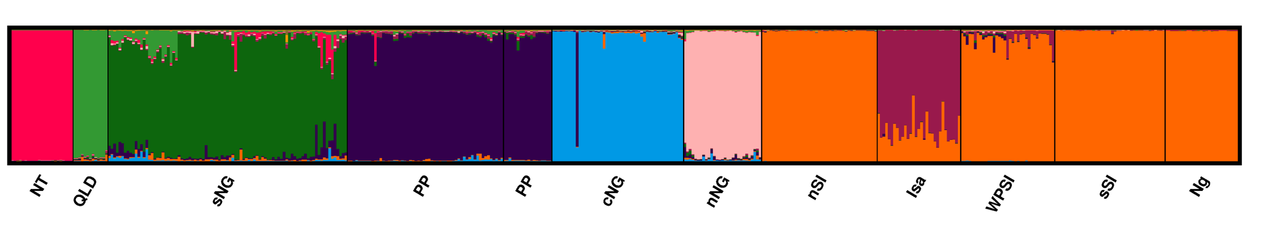

K=11 MinorCluster1

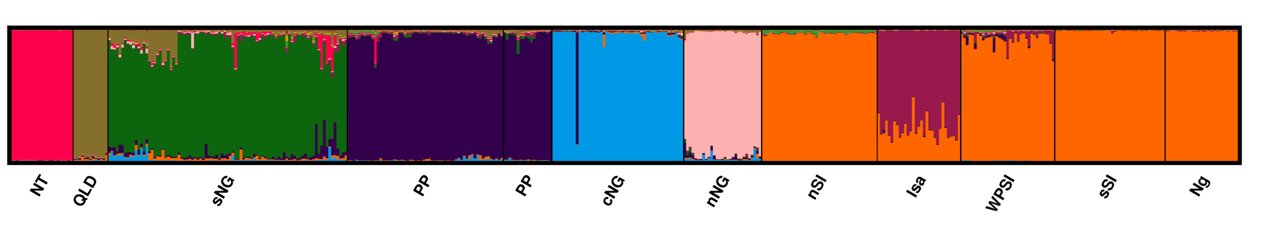

K=12 MinorCluster1

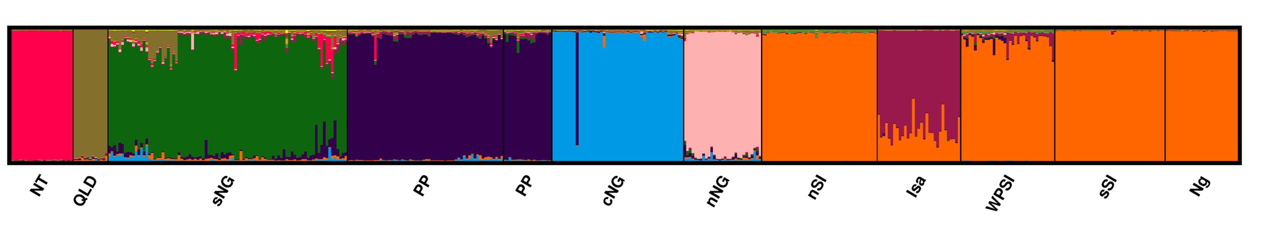

K=13 MinorCluster1

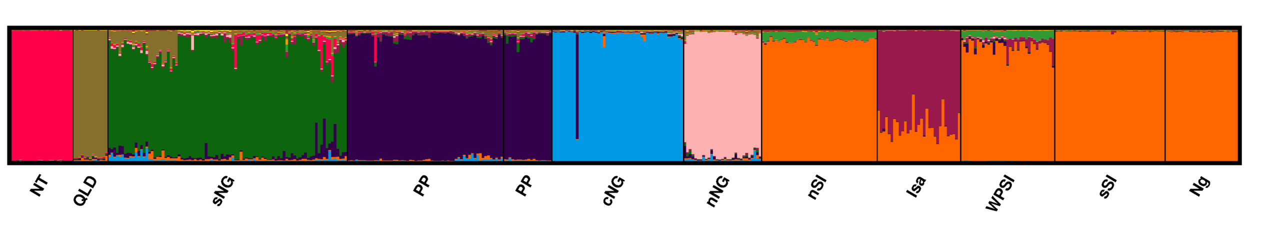

K=14 MinorCluster1

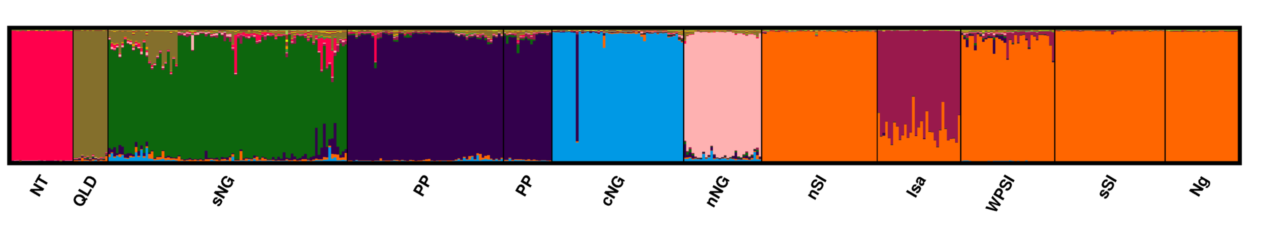

K=15 MinorCluster1

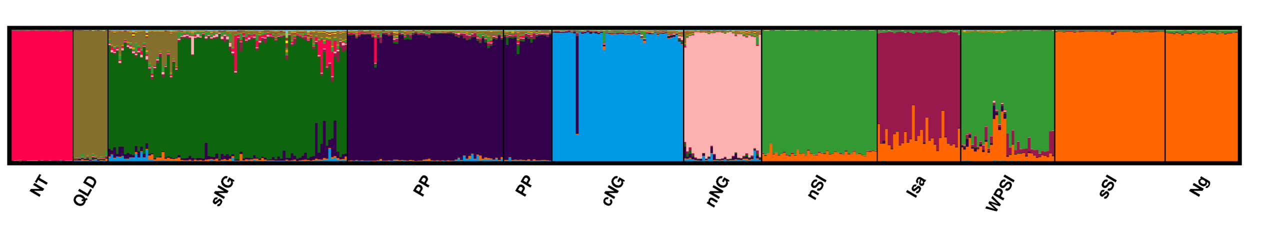

Division of runs by mode:

K=2 20/20
K=3 8/20, 6/20, 6/20
K=4 18/20
K=5 13/20, 6/20
K=6 6/20, 5/20, 4/20, 3/20, 2/20
K=7 10/20, 10/20
K=8 14/20, 3/20, 2/20
K=9 8/20, 7/20, 3/20, 2/20
K=10 12/20, 8/20
K=11 10/20, 10/20
K=12 13/20, 7/20
K=13 11/20, 9/20
K=14 11/20, 9/20
K=15 10/20, 10/20
